# Association mapping for cold tolerance in two large maize inbred panels

**DOI:** 10.1186/s12870-016-0816-2

**Published:** 2016-06-06

**Authors:** Pedro Revilla, Víctor Manuel Rodríguez, Amando Ordás, Renaud Rincent, Alain Charcosset, Catherine Giauffret, Albrecht E. Melchinger, Chris-Carolin Schön, Eva Bauer, Thomas Altmann, Dominique Brunel, Jesús Moreno-González, Laura Campo, Milena Ouzunova, Ángel Álvarez, José Ignacio Ruíz de Galarreta, Jacques Laborde, Rosa Ana Malvar

**Affiliations:** Misión Biológica de Galicia, Spanish National Research Council (CSIC), PO Box 2836080, Pontevedra, Spain; INRA, UMR de Génétique Végétale/Université Paris-Sud – CNRS – AgroParisTech, Gif-sur-Yvette, France; UMR INRA/USTL 1281 Stress Abiotiques et Différenciation des Végetaux cultivés, Péronne, France; Institute of Plant Breeding, Seed Science and Population Genetics, Universität Hohenheim, Stuttgart, Germany; Plant Breeding, Technische Universität München, Freising, Germany; Molecular Genetics, Leibniz Institute of Plant Genetics and Crop Plant Research (IPK), Gatersleben, Germany; INRA-VERSAILLES, Evry, France; Centro Investigacións Agrarias Mabegondo (CIAM), A Coruña, Spain; KWS SAAT AG, Einbeck, Germany; Estación Experimental de Aula Dei (CSIC), Saragossa, Spain; NEIKER-Instituto Vasco de Investigación y Desarrollo Agrario, Vitoria, Spain; INRA, Stn Expt Mais, F-40590 St Martin De Hinx, France

**Keywords:** GWAS, Maize, Cold tolerance, Chilling, QTL

## Abstract

**Background:**

Breeding for cold tolerance in maize promises to allow increasing growth area and production in temperate zones. The objective of this research was to conduct genome-wide association analyses (GWAS) in temperate maize inbred lines and to find strategies for pyramiding genes for cold tolerance. Two panels of 306 dent and 292 European flint maize inbred lines were evaluated *per se* and in testcrosses under cold and control conditions in a growth chamber. We recorded indirect measures for cold tolerance as the traits number of days from sowing to emergence, relative leaf chlorophyll content or quantum efficiency of photosystem II. Association mapping for identifying genes associated to cold tolerance in both panels was based on genotyping with 49,585 genome-wide single nucleotide polymorphism (SNP) markers.

**Results:**

We found 275 significant associations, most of them in the inbreds evaluated *per se*, in the flint panel, and under control conditions. A few candidate genes coincided between the current research and previous reports. A total of 47 flint inbreds harbored the favorable alleles for six significant quantitative trait loci (QTL) detected for inbreds *per se* evaluated under cold conditions, four of them had also the favorable alleles for the main QTL detected from the testcrosses. Only four dent inbreds (EZ47, F924, NK807 and PHJ40) harbored the favorable alleles for three main QTL detected from the evaluation of the dent inbreds *per se* under cold conditions. There were more QTL in the flint panel and most of the QTL were associated with days to emergence and ΦPSII.

**Conclusions:**

These results open new possibilities to genetically improve cold tolerance either with genome-wide selection or with marker assisted selection.

**Electronic supplementary material:**

The online version of this article (doi:10.1186/s12870-016-0816-2) contains supplementary material, which is available to authorized users.

## Background

Maize (*Zea mays* L.) is a tropical crop currently cultivated in high latitudes thanks to historical improvements of cold tolerance, reductions in growth cycle, and adaptation to long days [1]. Improved cold tolerance would allow earlier sowing dates and thus would enable escaping summer drought, pests and diseases [2]. Earlier sowing would also lead to longer vegetation periods, which can be used for biomass accumulation. Maize genotypes grown in temperate areas have moderate cold tolerance and previous studies have found only some genotypes with partial tolerance [3–6].

Since the advent of molecular markers and QTL studies, several reports have been published with limited impact on maize breeding for cold tolerance [7]. QTL reported for cold tolerance were associated with traits such as chlorophyll content or photosynthesis [5, 8–10]. Strigens et al. [11] carried out genome-wide association mapping for cold tolerance in a collection of maize inbred lines and obtained 19 QTL explaining between 5.7 and 52.5 % of the phenotypic variance for early growth and chlorophyll fluorescence. Due to the highly complex architecture of cold tolerance-traits, they proposed whole genome prediction approaches rather than classical marker assisted selection for improving chilling tolerance of maize.

Maize grown in cold areas of Europe is reported to stand low temperatures better than maize from other origins. Moreover, genotypes belonging to the European Flint germplasm showed better cold tolerance than those originating from the Corn Belt Dent [11, 12]. Previous reports found sources of cold tolerance in diverse collections of European germplasm [4, 6, 13–17]. The largest study for evaluation of cold tolerance was reported by Revilla et al. [4] who evaluated the same two large panels of maize inbred lines adapted to Europe for cold tolerance that was used for the present study. These authors found that the dent and flint germplasm most tolerant to cold temperatures were the Northern Flint D171 and the Iodent PH207 groups, respectively. They also concluded that models intending the prediction of final performance from traits scored in early developmental stages are not precise enough for breeding. Nevertheless, breeding for cold tolerance could be accomplished by combining inbreds from groups that can provide sources of favorable alleles for cold tolerance. The evaluation method and the traits used for assessing cold tolerance at early stages of development have been defined according to our previous experience [4] taking into account traits that estimate cold tolerance for the subsequent steps of the heterotrophic stage from germination to endosperm depletion. According to previous information [3], the main detrimental effects of cold conditions at early stages of maize development are delayed emergence, reduced chlorophyll content and efficiency of photosystem II, and decreased early vigor and biomass synthesis. Therefore, we have recorded data related to those features that can accurately be measured with large numbers of genotypes.

The objective of this study was to carry out genome wide association analyses for cold tolerance in two large panels of maize inbred lines and to suggest possible strategies for breeding new genotypes with improved cold tolerance.

## Methods

### Plant material

Two panels of 306 dent and 292 flint maize inbred lines [4, 18] representing the breeding germplasm adapted to European agro-climatic conditions were evaluated *per se* and as testcrosses [4]. The panels were built from the collections of Spanish, French, and German breeders involved in this research. They come from Western Europe and the USA. The inbreds are public and have been released throughout the history of maize breeding. The seed used in this study was produced by the INRA (France), the Technical University of Munich (Germany) and the Spanish institutions CSIC, NEIKER and CIAM. The dent inbreds were crossed to the flint tester UH007 and the flint inbreds to the dent tester F353 in a winter nursery in 2010 in order to evaluate testcross performance using each tester as the male parent and the inbreds of the panels as female parents [4].

### Growth chamber trials

We used a cold chamber of 20 m^3^ built inside a laboratory with modulated panels, isolated with injected polyurethane. The 598 flint and dent inbreds were evaluated *per se*, along with six checks (C105, CO109, D152, EA1027, F816, FP1). Dent and flint panels were evaluated separately in adjacent trials under cold and control conditions. After inbreds *per se* evaluation, testcrosses were evaluated for cold and for control conditions. Evaluations of inbreds *per se* and testcrosses in control and cold conditions were made in consecutive runs. Each trial followed a randomized complete block design with six replications [4].

Maize kernels were planted in a multi-pot trays; using one cell for each kernel. Each cell had a surface of 3 cm × 2.5 cm and 5 cm depth filled with sterilized peat (Gramoflor GmbH & Co. KG, Vechta, Germany). Six plants per inbred or testcross were used in each run of the growth chamber as there were six repetitions with one plant per replication in each trial. The experiments were watered after planting; afterwards the trials were watered as needed. Temperature and light conditions for the cold experiments were 14 °C/14 h with light and 8 °C/10 h in the dark. In the control experiments, plants were grown at 25 °C/14 h light and 20 °C/10 h dark. Cool light was provided by seven very high output fluorescent lamps per shelf with a photosynthetic photon flux of 228 μmol m^−2^ s^−1^. Distance between shelves and fluorescent lamps was 0.5 m.

In every trial, data were recorded for 1) number of days from sowing to emergence, 2) relative leaf chlorophyll content (SPAD units) in the second leaf, using a hand-held CCM-200 Chlorophyll Content Meter (Opti-Sciences, Tyngsboro, Massachusetts, USA), 3) quantum efficiency of photosystem II (ΦPSII) recorded in the second leaf by using a portable OS-30p Chlorophyll Fluorometer (Opti-Sciences, Tyngsboro, Massachusetts, USA) [4]. For inbreds *per se* we scored early vigor using a visual scale from 1 = weak plants to 9 = vigorous plants. For testcrosses, dry weight was determined by weighing the plants after drying them in an oven at 80 °C during 5 days.

### Statistical analysis

All inbreds were genotyped with the Illumina MaizeSNP50 BeadChip that includes 49,585 SNPs covering all 10 maize chromosomes [19]. According to these authors, the design of this library started with 839,350 SNP derived from the first generation haplotype map (Panzea set.), a collection of markers arising between B73 and Mo17 provided by Syngenta, and SNPs chosen from comparative sequencing of B73 and F2, provided by INRA, as well as SNPs collected from various other published marker sets [19]. They eliminated duplicated SNPs and those SNPs that contained nearby known SNPs in both flanking sequences, followed by four further stages of selection aiming at optimizing coverage of the genome and even spacing throughout the genome. Data were filtered to exclude SNPs with more than 20 % missing values and less than 5 % minor allele frequency. Heterozygote genotype calls were considered as missing data. 42,214 and 35,963 SNPs in the -Dent and Flint panels, respectively were used for GWAS. Genotypic data are available at Rincent et al. [18].

We used the genotyping matrix and a genetic kinship matrix (K) described earlier by Rincent et al. [18]. Best linear unbiased estimators (BLUEs) for inbred lines and testcrosses were calculated for each panel with the SAS mixed model procedure (PROC MIXED) in SAS software version 9.3 [20] considering inbred lines or testcrosses as fixed effects and replications as random effects.

Genome-wide association analysis based on mixed linear model (MLM) was performed with software Tassel 4.1.26 [21]. The MLM used by Tassel was

$$ \mathbf{y}=\mathbf{X}\boldsymbol{\upbeta } +\mathbf{Z}\mathbf{u}+\mathbf{e} $$

where **y** is the vector of phenotypes (BLUEs), **β** is a vector of fixed effects, including the SNP marker tested, **u** is a vector of random additive effects (inbred lines), **X** and **Z** represent matrices of 1 s and 0 s related to **β** and **u** respectively, and **e** is a vector of random residuals. The variance of random line effects was modeled as $Var(u)=K\;{\sigma}_a^2$, where **K** is the *n* × *n* matrix of pairwise kinship coefficients and ${\sigma}_a^2$ is the estimated additive genetic variance [22]. Restricted maximum likelihood estimates of variance components were obtained by using the optimum compression level (compressed MLM) and population parameters previously determined (P3D) options in Tassel [23]. The optimum compression level option reduces the computation demand by clustering the (*n*) total individuals into (*s*) groups based on their realized genomic relationships, allowing the original **K** matrix to be replaced by a smaller relationship matrix.

The statistical significance threshold was set to 0.05/M_eff_, which corresponds to a Bonferroni correction on M_eff_ tests, M_eff_ being the number of independent tests estimated [24]. We used the same threshold as Rincent et al. [18] because they used the same sets of lines. They evaluated 3638 and 3527 independent tests in the Dent and Flint panels respectively, which led to a -log_10_ (P-value) threshold of 4.9 in both panels. Significant SNPs separated by less than 700 kb were considered as a single QTL for the interpretation of the results. Likewise, if SNP1 was linked to SNP2, and SNP 2 was linked to SNP 3, then we considered SNPs 1, 2 and 3 the same QTL although SNP 1 and SNP 3 differed by 700 kb. We examined a 700 kb region left and right of each significant SNP in order to identify candidate genes of interest by use of the MaizeGDB genome browser [25].

Local linkage disequilibrium (LD)(r2) among markers in an 1500 kb interval surrounding the significant SNPs and common haplotype patterns were assessed in Haploview version 4.2 [26]. Haplotype blocks were defined with the confidence interval method of Gabriel et al. [27]. Only SNPs with a MAF ≥ 0.05 and less than 0.20 missing data were used to estimate LD.

Heritability (*ĥ*^2^) for each panel (dent, flint), each condition (cold and control) and inbreeding level (inbreed, hybrid) were estimated for each trait on a family-mean basis as described by Holland et al. [28].

## Results and discussion

### Association analyses

The compressed mixed linear model analyses for cold tolerance traits reduced the genetic effects by a compression level from 1 to 18.6, being lowest for early vigor/early dry weight followed by days to emergence, and highest for ΦPSII followed by chlorophyll content, although in most cases compression levels were in the range 1 to 2 (Additional file 1: Table S1). Compression levels were made for grouping inbred lines and for making the subsequent analyses with groups, taking into account the similarities among inbreds within the panels. Random genetic variability was not uniformly distributed for diverse traits and evaluation conditions and the number of individuals per group was variable as well, indicating that residual random variability was inconsistently distributed within groups.

According to Zhang et al. [23] the best control of the false positive rate for the validation of the compressed mixed linear model approach was when the compression levels were within a range of 1.5 to 10 [28]. Therefore, the control of false positives by our model is efficient for days to emergence and also for early vigor/early dry weight. Focusing on inbreds *per se* under control conditions, we should note that compression level was less than 1.5 for all traits except days to emergence for flint panel and early vigor for dent panel. Background genetic effects modeled by K ranged from 2 % of the total phenotypic variation to 79 % in lines and from 4 to 59 % in hybrids. The proportion was higher for flint than for dent panel, for inbreds *per se* than for hybrids, and for ΦPSII and early vigor. Finally, the proportion was similar for evaluations under cold and control conditions, (Additional file 1: Table S1).

In general, traits showed intermediate heritability values (*h*^2^ around 0.50) except for days to emergence. For this trait, low heritability value was obtained (*h*^2^ around 0.25, Additional file 1: Table S1). For ΦPSII, inbreeds showed higher heritability values than hybrids. Heritability values were similar for dent and flint panels and for control and cold conditions. We expect higher genetic variability under cold conditions and higher error variance therefore heritability was similar under both evaluation conditions.

### QTL analyses

The numbers of markers adequate for GWAS analysis depends of the rate of linkage disequilibrium (LD) decay, the panel diversity, and the objective of GWAS analysis. LD decays fast and the diversity is large in maize, so a high number of markers should be used especially if the approaches is looking for candidate genes. However, both panels (dent and flint) are composed of lines adapted to Europe and therefore we expect less variability than in the American panel where there are tropical and temperate lines. Besides, the objective is looking for QTL associated to cold tolerance traits to explore new breeding possibilities rather than looking genes related to cold tolerance. Therefore, for these panels the Illumina MaizeSNP50 BeadChip is adequate [29].

QTL analyses were made separately for each panel (dent and flint), inbreeding level (inbreds *per se* and testcrosses) and environmental conditions (cold and control), although we focused mainly on the analyses of inbreds *per se* under cold conditions (see below). Number of panel lines used for GWAS is highly important for the mapping power. For traits regulated by large number of loci with small effect increasing sample size will improve power. However, it will often increase genetic heterogeneity and could reduce the detection power especially for traits that are important for adaptation like cold tolerance traits [30]. Besides, it is important to notice that most European flint inbreds have some historical and genetic relationships and most of them come from germplasm that has been adapted to European conditions for several centuries. Conversely, the dent panel includes genotypes that have been introduced in Europe during the last decades without consistent historical or genetic relationships among groups [4]. Therefore, flints and dents should be analyzed separately in order to respect the genetic structure of the genotypes.

**Fig. 1:**
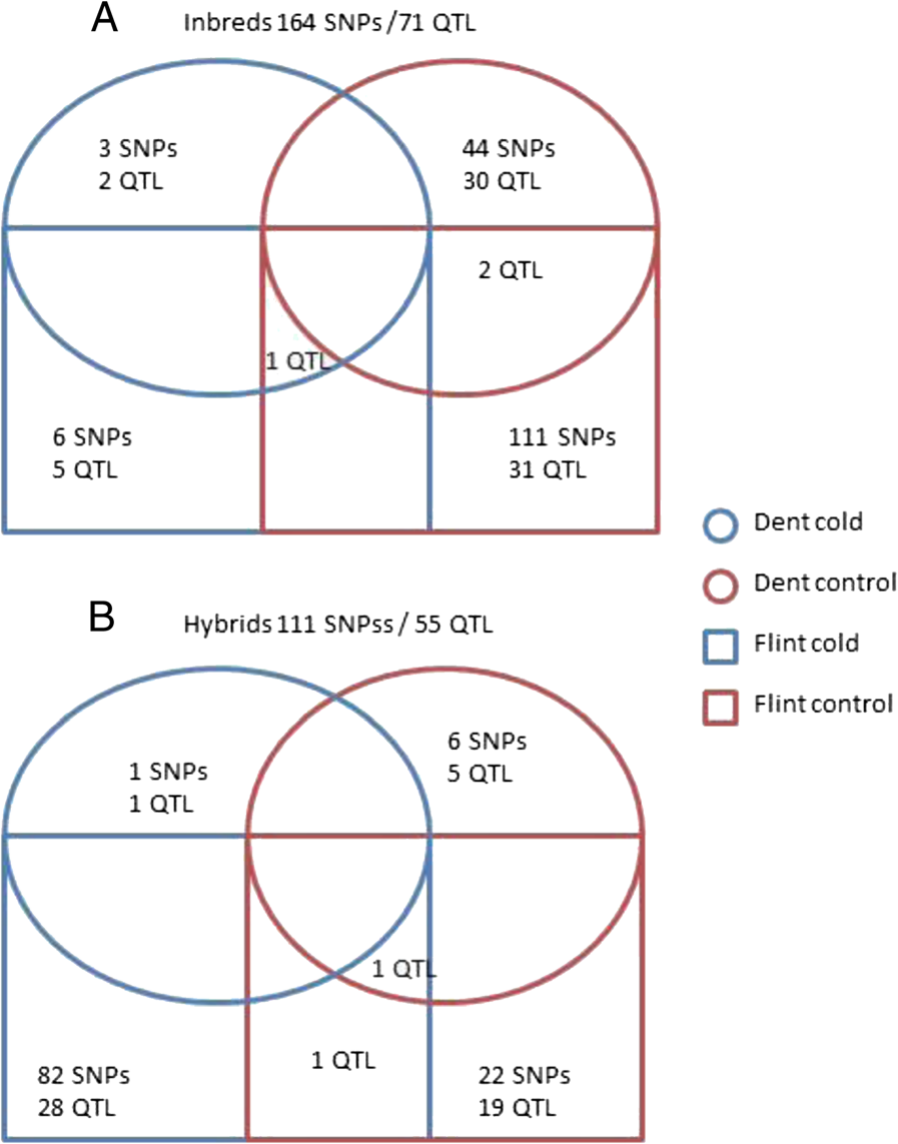
Significant SNPs and QTL associated to cold tolerance traits in two association panels of maize. Inbred lines were evaluated *per se* (**a**) and in testcrosses (**b**) under control and cold conditions

Altogether, we found 275 SNPs significantly associated to any trait, most of them were found for the inbreds evaluated *per se* (164 significant associations/71 QTL) (Fig. 1). The higher number of significant QTL found in inbred lines, compared to testcrosses could be due to the masking effect of the inbred tester used for producing testcrosses, as pointed out by previous reports [4]. Most of the significant associations (117) were found in the flint panel, probably because there was more variability for traits related to early plant development among flint inbred lines than among dent lines [4]. Finally, most QTL (90) were identified under control conditions (38 in cold conditions), presumably because experimental errors were higher under stress conditions than under optimum conditions [3]. Similarly, Strigens et al. [11] found more QTL under control conditions than under cold conditions. However, Strigens et al. [11] found only 19 QTL under cold conditions probably because they evaluated a panel with fewer lines (375 dent and flint inbred lines) than our two panels together (306 dent and 292 flint inbred lines).

Evaluations of dent testcrosses under cold conditions identified one QTL for days to emergence on chromosome 3 (chr3) and five QTL under control conditions on chr4, 5, 7 and 10 (Fig. 1, Additional file 2: Table S2). For ΦPSII, dent testcrosses yielded only one QTL on chr10 under control conditions and none under cold conditions. Flint testcrosses evaluated under cold conditions had 29 QTL for days to emergence located on all chromosomes except chr5, while under control conditions there were 20 QTL on all chromosomes. For ΦPSII, flint testcrosses had one QTL on chr4 under cold conditions, and none under control conditions. For early dry weight there was only one QTL on chr9 for flint testcrosses under control conditions.

Evaluations of dent inbreds *per se* under control conditions identified 21 SNPs (16 QTLs) on chr1, 2, 3, 5, 7, 8, and 10 for days to emergence, and for ΦPSII there were 23 SNPs (18 QTL) on chr1, 2, 3, 5, 7, 9, and 10. Six significant SNPs were found in both traits (Additional file 2: Table S2). Flint inbreds evaluated *per se* under control conditions revealed 11 SNPs (8 QTL) for days to emergence on chr6, 7, 8, 9, and 10, and 100 SNPs (26 QTL) on chr3, 4, 5, 9, and 10 for ΦPSII.

**Fig. 2:**
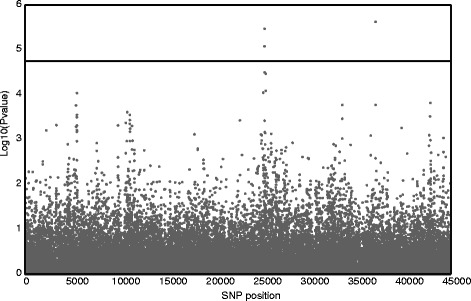
GWAS results for early vigor in the Flint panel. The graph represents -log_10_(*P*-values) of the 35963 SNPs tested. The line shows the significant threshold of -log_10_(*P*-values)

Nine SNPs (8 QTL) were significantly associated to cold tolerance-related traits for the inbred lines evaluated *per se* under cold conditions, three of them for the dent panel and six for the flint panel (Table 1). The traits with significant SNPs were early vigor (4 SNPs), three of them for the Flint panel (Fig. 2), days to emergence (1), ΦPSII (2) and SPAD (2) that were located on chr1, 3, 4, 5 and 7. The additive effect indicates that the major allele provides increased cold tolerance for the QTL of days to emergence, both QTL of ΦPSII and one QTL of early vigor and less cold tolerance for both QTL of SPAD and three QTL of early vigor. The frequency of the alleles at each QTL was moderate except for some cases such as PZE-101084685 with a frequency ratio of 27/213. Finally, these QTL explained a proportion of phenotypic variance between 8 and 14 %, a range similar to that reported in previous publications of QTL for cold tolerance [11].

**Table 1:** SNPs significantly associated to early growth-related traits, from association analyses in two panels of maize

Inbred panel	Chromosome	Position	SNP	*P-*value	SNP alleles	Additive^a^ effect	N^b^	*R* ^2c^
Days to emergence								
Flint	3	145737736	PUT-163a-78121249-4393	3.3 × 10^−6^	T/C	0.67	201/40	0.11
Chlorophyll content (SPAD)								
Dent	1	201477347	PZE-101159230	1.4 × 10^−5^	C/T	0.34	234/57	0.08
Dent	4	172689894	SYN2344	1.5 × 10^−5^	A/G	0.18	157/131	0.08
ΦPSII^d^								
Flint	1	73380804	PZE-101084685	8.1 × 10^−6^	A/C	59.5	27/213	0.10
Flint	4	20738948	SYN24026	8.0 × 10^−6^	T/G	56.6	38/203	0.09
Early vigor^e^								
Dent	1	110914351	PZE-101106625	1.2 × 10^−5^	C/T	0.05	231/62	0.08
Flint	5	27247368	PZE-105041198	8.3 × 10^−6^	C/T	0.21	62/179	0.11
Flint	5	27857856	PZE-105041551	3.4 × 10^−6^	C/T	0.21	123/97	0.14
Flint	7	153421340	PZE-107098206	2.4 × 10^−6^	C/T	0.21	121/118	0.10

^a^The additive effect was calculated as half the difference between the mean of the homozygotes for the minor and the mean of the homozygotes for the major allele

^b^Number of lines with each allele

^c^R^2^, proportion of total line mean variance explained by SNP as computed by Tassel software

^d^ΦPSII: Quantum efficiency of PSII

^e^Early vigor: subjective score from 1 = weak plants to 9 = vigorous plants

For significant SNPs under cold conditions, an analysis of variance was performed to test SNP × environment interaction. SNPs detected in the dent panel did not show significant interactions with environment. On the other hand, the interactions were significant for five of six significant SNPs detected in the Flint panel. However, the interactions are range type because 1) SNP alleles did not differ under control conditions, or 2) the favorable allele is the same under both conditions but the difference between the two alleles is significantly higher under cold conditions (data not shown). This shows again the different behavior of Flint and Dent panels under cold conditions.

**Fig. 3:**
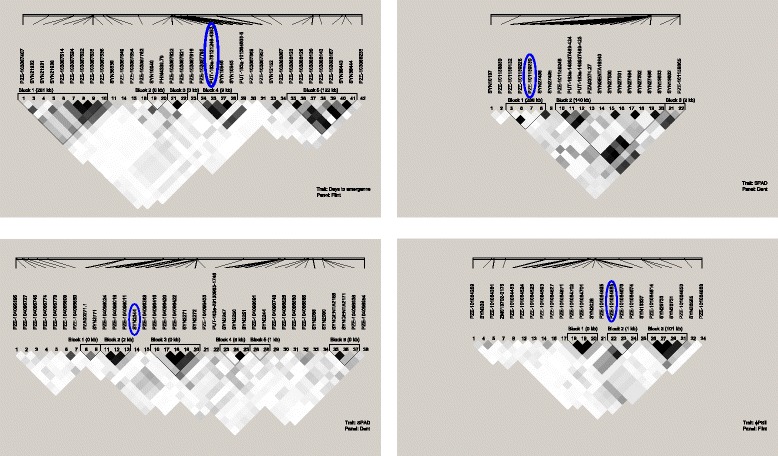
Local LD, measured as r^r^ values between pairs of SNPs (*white*, r ^2^ = 0; *shades of gray* = 0 < r^2^<, 1; *black* r^2^ = 1), and haplotype blocks for a 1.4 -kb genomic region that surrounds significant SNPs associated to cold tolerance traits in two association panels of maize inbred lines evaluated under control and cold conditions

Local LD in a 1400 kb interval surrounding the significant SNPs shows the rapid decay of LD between pairs of markers [31]. In fact, 3 of 9 significant SNPs cannot be included in haplotype blocks defined with the confidence interval method [31] (Fig. 3). Composition haplotype groups vary from 1 to 8 SNPs. For days to emergence haplotypes with favorable alleles are found in 80 % of the lines while for chlorophyll content (SNP PZE-101159230) haplotype with all favorable alleles are only in 20 % of genotypes. Haplotype with all favorable alleles for early vigor is found in a frequency of less than 10 % for both SNPs PZE-101106625 and PZE-107098206 (Table 2)

**Table 2:** Haplotype effects significantly associated to early growth-related traits, from association analyses in two panels of maize

SNP	Trait	Panel	Haplotype	Haplotype	Haplotipe	Additive effects
				Frecuency	Mean	Average
PUT-163a-78121249-4393	Days to emergence	Flint	G,C,C,A	15	11.80 ± 0.23	1.28
			T,T,A,G	80	11.66 ± 0.09	0.16
PZE-101159230	Chlorophyll content (SPAD)	Dent	G,T,C,C	51	4.54 ± 0.17	−1.26
			A,T,T,C	20	5.06 ± 0.27	−0.90
			G,T,C,T	18	4.25 ± 0.24	−1.38
			G,C,C,C	11	4.06 ± 0.36	−1.98
SYN2344	Chlorophyll content (SPAD)	Dent	G	44	4.76 ± 0.17	−3.04
			A	54	4.19 ± 0.14	−3.42
PZE-101084685	ΦPSII	Flint	CCA	46	442 ± 10	238
			CTG	33	450 ± 13	228
			ATG	13	322 ± 29	189
SYN24026	ΦPSII	Flint	C,A,G,G	49	465 ± 10	55
			C,A,G,A	27	418 ± 12	59
			C,G,T,G	15	333 ± 21	−2
PZE-101106625	Early vigor	Dent	T,C,G,G,G,C,C,A	39	3.99 ± 0.07	1.10
			C,C,T,A,A,T,C,G	16	4.04 ± 0.10	1.02
			T,C,G,G,A,C,T,A,	22	4.03 ± 0.47	1.10
PZE-105041198	Early vigor	Flint	T	73	3.95 ± 0.04	−1.19
			C	26	3.53 ± 0.07	−1.61
PZE-105041551	Early vigor	Flint	C	51	3.61 ± 0.05	0
			T	38	4.07 ± 0.05	0.42
PZE-107098206	Early vigor	Flint	CT	51	3.62 ± 0.05	−0.19
			TT	42	4.03 ± 0.05	0.02

The most favorable allele for each SNPs is underlined. haplotypes are considered when the frequency is greater than 10 %

Candidate genes were chosen based on the genomic sequence of the maize inbred line B73 [32] within an interval of 100 kbp wide flanking regions upstream and downstream from the significant SNP. We identified the closest candidate gene for the QTL in flint associated to PUT-163a-78121249-4393 on chr3 for days to emergence with an intracellular signal transduction function and there were eight possible candidate genes in the interval (Table 3). However, as in the QTLs, candidate genes were also looked for in a wider region of 1400 kb significant around the SNP (Additional file 3: Table S3). The closest candidate gene among the five genes close to the QTL in dent for SPAD on chr1 (associated to PZE-101159230) has a protein heterodimerization activity. For the QTL in dent for SPAD on chr4 (associated to SYN2344), there were four candidate genes and the closest one has a starch synthase activity; Strigens et al. [11] also found a QTL close to this position for one of the environments where they evaluated a panel of maize inbred lines for cold tolerance. The closest candidate gene between the two genes close to the QTL in flint for ΦPSII on chr1 (PZE-101084685) has an oxidoreductase activity. There were three candidate genes around a QTL in dent for early vigor on chr1 (PZE-101106625) and the closest one has an unknown activity. The only candidate gene in the QTL interval in flint for early vigor on chr5 (PZE-105041551) has a sequence regulator activity. A second QTL in flint for early vigor on chr5 is only around 600 kb apart (PZE-105041198) and the closest candidate gene among the two in this QTL interval has a protein kinase activity. Finally, the QTL in flint for early vigor located on chro7 (PZE-107098206) had nine candidate genes in the interval and the closest one has an unknown activity; but near this location, Strigens et al. [11] found a QTL for ΦPSII, a trait related to early vigor, that was associated to a subtilisin-like protease precursor and a putative ethylene-insensitive3-like protein. Several of the candidate genes identified have unknown function in maize and for many others, the annotation is based on homology to other species. These candidate genes could be studied in subsequent research projects in order to understand their role in the genetic regulation of cold tolerance.

**Table 3:** Candidate genes from the association analyses for cold tolerance traits in two association panels of maize

Chr.	SNP ID and position of significant SNP (bp)	Gene ID	Start (bp)	Stop (bp)	Function/Subcellular localization
Days to emergence					
3	PUT-163a-78121249-4393 145737736	GRMZM2G061206	145636198	145639549	Antiporter
		GRMZM2G061127	145642310	145643935	Proteolysis/Chloroplast stroma localization
		**GRMZM2G174274**	**145762729**	**145764119**	**Signal transduction/Intracellular**
		GRMZM2G174249	145763792	145766257	Carbohydrate, response to wounding/Cell wall vacuole
		GRMZM2G174221	145771397	145774255	Vacuole
		GRMZM2G174196	145774369	145777597	Protein binding
		GRMZM2G174137	145786387	145795884	Catalytic activity
		GRMZM2G074241	145796992	145799980	ATPase activity/Membrane
		GRMZM2G375807	145817237	145825052	ATPase activity/Membrane
Chlorophyll content (SPAD)					
1	PZE-101159230 201477347	GRMZM2G419024	201439240	201443106	Triosephosphate isomerase activity
		**GRMZM5G899800**	**201502394**	**201505169**	**Protein heterodimerization activity**
		GRMZM2G416069	201513870	201519179	Protein heterodimerization activity/Cohesin complex
		GRMZM2G115730	201519254	201520692	Unknown
		GRMZM2G115750	201522268	201525873	Receptor/Membrane, ER, Golgi
4	SYN2344 172689894	**GRMZM2G130043**	**172635729**	**172706662**	**Starch synthase**
		GRMZM2G130002	172714527	172719310	Protein binding/Plasma membrane
		GRMZM2G129979	172721820	172724795	G10 protein/Nucleous
		GRMZM2G178398	172777639	172783737	Epsin like protein
ΦPSII^a^					
1	PZE-101084685 73380804	GRMZM2G172244	73387395	73390846	Unknown
		**GRMZM2G171420**	**20758772**	**20762634**	**Unknown**
4	SYN24026 20738948	GRMZM2G171394	20762995	20764457	Rapid alkalinization factor
		GRMZM2G078143	20795669	20799891	Glycine hydroxymethyltransferase/Plasma membrane
Early vigor^b^					
1	PZE-101106625 110914351	GRMZM2G084825	110955450	110958921	Protein serine threonine kinase activity
		GRMZM2G154216	110981685	110983586	Transferase activity of acyl groups (no amino-acyl)
		GRMZM2G341036	27150769	27152027	Unknown
5	PZE-105041198 27247368	**GRMZM2G102862**	**27218204**	**27224147**	**Protein kinase activity**
		**GRMZM2G405090**	**27787706**	**27788723**	**Putative uncharacterized protein**
5	PZE-105041551 27857856	GRMZM2G127510	153320097	153324724	Nucleotide binding
7	PZE-107098206 153421340	GRMZM2G127499	153326363	153329791	Unknown
		GRMZM2G429396	153330870	153331777	Response to high light intensity/Cytoplasm
		GRMZM2G124794	153413756	153414250	Deoxyribodipyrimidine photo-lyase activity
		GRMZM2G423478	153416453	143416869	Unknown
		**GRMZM2G180027**	**153457846**	**153464555**	**Unknown**
		GRMZM2G480480	153464547	153475981	Protein binding
		GRMZM2G180080	153485164	153487062	Nucleotide binding
		GRMZM2G180082	153490387	153492351	Oxidoreductase activity, acting on paired donors, with incorporation or reduction of molecular oxygen

Genes in an 200 kb interval surrounding the significant SNPs are listed with their identifier. The gene closest to the significant SNP is indicated in bold^a^ΦPSII: Quantum efficiency of PSII

^b^Early vigor: subjective score from 1 = weak plants to 9 = vigorous plants

### Breeding strategies for improving cold tolerance

There are few significant QTL associated with cold tolerance, which explain a small proportion of the phenotypic variance for cold tolerance characters. Therefore, it would be interesting to build synthetics of lines with favorable alleles of significant QTL for cold tolerance. These synthetics could be the base material to start phenotypic or genomic selection programs aiming at the development of lines with improved cold tolerance. To choose the lines, we should take into account significant SNPs and if they are linked to other SNPs forming haplotype groups. There are 47 flint inbreds with favorable alleles for all six QTL associated with cold tolerance. However, there are not inbreds with all favorable alleles for all SNPs of haplotypes groups. It is because the combination of favorable alleles for the SNP SYN24026 group, associated to ΦPSII, was shown only by CH19-1, UHF07721H and UHL016. These inbreds did not have favorable alleles for other traits but they should be part of the synthetic to keep favorable alleles for cold tolerance. The best inbreds were EZ33 and Ia2132 with 14 favorable alleles out of the 15 significant SNPs and haplotype groups (Additional file 4: Table S4). On the other hand, among the 47 inbreds six belonged to the group Northern Flint, 27 to the Northern Flint family of D171, and one to the Northern Flint family of FV7. The remaining 13 selected inbreds belonged to No-Northern Flint groups according to the grouping revealed in our previous study [4]. Among the 47 selected inbreds, four (D171, EZ33, Ia2132 and UH1494) had also the favorable alleles for the three SNPs most significantly associated in the testcross trials under cold conditions (Additional file 4: Table S4). These four inbreds are unrelated and could be the base of a Northern Flint synthetic with the best combination of cold tolerance alleles and a wide genetic base, considering the diversity available within the Northern Flint race. CH19-1, UHF07721H and UHL016 are also Northern Flint and should be included in the synthetic to increase ΦPSII under cold conditions.

Concerning the dent panel, four of the inbreds (EZ47, F924, NK807 and PHJ40) carried the favorable alleles for the three QTL detected for inbred lines *per se* evaluated under cold conditions (Additional file 5: Table S5) and all of them had favorable alleles for all SNPs of haplotype group for SPAD and 6/8 for haplotype group for early vigor. These inbreds were unrelated and belonged to a mixed group, except F924 that belongs to the Stiff Stalk pool. Besides these four inbreds, there were eight inbred lines (B37, EZ11A, EZ46, F618, F918, FV317, N6, and PHG80) that had two favorable alleles for the QTL of inbreds *per se* and for two QTL for the testcrosses. Most of the inbreds carrying two favorable alleles belong to the “mixed” group. Especially interesting were EZ11A and EZ46 because they had one favorable allele for early vigor that was not present in previously selected lines. However, to complete the favorable haplotype for early vigor we need the favorable allele for the first SNP of the group. AS5707, B103, F904 or PHK29 could be donor of this allele, besides they had two favorable alleles for the QTL of inbreds *per se*.

Among the inbreds previously selected for cold tolerance *per se* [4], in the present work the flint inbreds FV71 and UH006, and the dent inbreds LH85 and FV335 were included in the set of selected inbreds based on genotype (Additional file 4: Table S4 and Additional file 5: Table S5). Considering the groups, the northern flint group D171 and the Iodent group PH207 were among the selected groups based on both phenotype and genotype.

## Conclusions

From this study, we draw four conclusions. (1) The use of two large panels of inbreds from the dent and flint genepools enabled identifying the largest number of QTL for cold tolerance ever published. (2) More QTL for cold tolerance were found in the European flint panel than in the dent panel. (3) Most of the QTL were associated with days to emergence and efficiency of photosystem II. (4) These results open new possibilities for improving cold tolerance either with genome-wide selection or with marker-assisted selection in maize breeding.

## Abbreviations

SSR, simple sequence repeats; GWAS, genome-wide association analyses; SNP, single nucleotide polymorphism; QTL, quantitative trait loci; BLUE, Best linear unbiased estimators; MLM, mixed linear model.

## Additional files

Additional file 1: Table S1. Summary of the compression mixed linear model analysis for cold tolerance traits in two panels of dent and flint maize inbred lines evaluated in a cold chamber under cold and control conditions *per se* and as testcrosses, and number of SNPs declared as significantly associated to each trait. (DOC 372 kb) Additional file 2: Table S2. SNPs declared as significantly associated to cold tolerance-related traits, chromosome location, *P*-Value, allele effect estimates, proportion of total variance explained by the SNPs from the association analyses for cold tolerance traits in two association panels of maize inbred lines evaluated in test-crosses under cold and control conditions and *per se* under control conditions. (DOCX 47 kb) Additional file 3: Table S3. Candidate genes from the association analyses for cold tolerance traits in two association panels of maize. Genes in a 1400 kb interval surrounding the significant SNPs are listed with their identifier. The gene closest to the significant SNP is indicated in bold. (DOCX 34 kb) Additional file 4: Table S4. Flint inbred lines with the favorable allele for the SNPs significantly associated to cold tolerance-related traits and allele composition for the significant SNPs with highest level of signification from the testcross trials of flint inbreds. (DOCX 36 kb) Additional file 5: Table S5. Dent inbred lines with the allele composition for the SNPs significantly associated to cold tolerance-related traits and allele composition for the significant SNPs with highest level of signification from the testcross trials of flint inbreds. Data: Best linear unbiased estimates (BLUEs) of early growth-related traits used for association analyses for cold tolerance traits in two association panels of maize inbred lines evaluated *per se* and hybrids under control and cold conditions. (DOCX 30 kb)
