# Supplementary material for: Association mapping for cold tolerance in two large maize inbred panels
Source: BMC Plant Biol. 2016 Jun 6;16:127. doi: 10.1186/s12870-016-0816-2 (PMC4895824; doi:10.1186/s12870-016-0816-2)
Supplement: Additional file 1: Table S1. — Summary of the compression mixed linear model analysis for cold tolerance traits in two panels of dent and flint maize inbred lines evaluated in a cold chamber under cold and control conditions per se and as testcrosses, and number of SNPs declared as significantly associated to each trait. (DOC 372 kb) [file 12870_2016_816_MOESM1_ESM.doc]

| Table S1. Summary of the compression mixed linear model analysis for cold tolerance traits in two panels. Dent and flint maize inbred lines were evaluated in a cold chamber under cold and control conditions *per se* and as testcrosses,  heritability on a line mean-basis, and number of SNPs declared as significantly associated to each trait | | | | | | | | | | | |
| --- | --- | --- | --- | --- | --- | --- | --- | --- | --- | --- | --- |
| Inbred  panel | Trial  conditions | | Inbreeding  level | n^a^ | s^b^ | Compression  level^c^ | (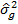)^d^ | (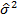)^e^ | 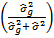^f^ | *h* ^2^± se | Significant SNPs |
|  | | Days to emergence: 141 SNPs | | | | | | | | | |
| Dent | Cold | | *Per se* | 295 | 231 | 1.28 | 0.471 | 1.400 | 0.25 | 0.10±0.02 | 21 |
| Dent | Control | | *Per se* | 299 | 141 | 2.12 | 6.860 | 3346.8 | 0.002 | 0.37±0.06 | 11 |
| Flint | Cold | | *Per se* | 242 | 114 | 2.12 | 0.692 | 1.325 | 0.34 | 0.24±0.07 | 0 |
| Flint | Control | | *Per se* | 244 | 234 | 1.04 | 0.003 | 0.016 | 0.15 | 0.13±0.08 | 1 |
| Dent | Cold | | Hybrid | 304 | 220 | 1.38 | 0.064 | 1.025 | 0.06 | 0.28±0.07 | 5 |
| Dent | Control | | Hybrid | 305 | 144 | 2.12 | 0.020 | 0.072 | 0.22 | 0.00±0.00 | 25 |
| Flint | Cold | | Hybrid | 243 | 243 | 1 | 0.553 | 0.634 | 0.47 | 0.20±0.08 | 1 |
| Flint | Control | | Hybrid | 243 | 110 | 2.21 | 0.019 | 0.050 | 0.28 | 0.24±0.11 | 77 |
|  | | SPAD^g^: 2 SNPs | | | | | | | | | |
| Dent | Cold | | *Per se* | 295 | 256 | 1.15 | 1.058 | 2.176 | 0.32 | 0.40±0.06 | 0 |
| Dent | Control | | *Per se* | 299 | 244 | 1.23 | 2.215 | 8.778 | 0.20 | 0.64±0.04 | 0 |
| Flint | Cold | | *Per se* | 242 | 13 | 18.6 | 1.965 | 5.334 | 0.26 | 0.52±0.05 | 2 |
| Flint | Control | | *Per se* | 244 | 220 | 1.11 | 8.283 | 9.878 | 0.45 | 0.60±0.04 | 0 |
| Dent | Cold | | Hybrid | 304 | 248 | 1.23 | 0.968 | 1.287 | 0.42 | 0.60±0.04 | 0 |
| Dent | Control | | Hybrid | 304 | 43 | 7.07 | 3.163 | 21.374 | 0.13 | 0.24±0.10 | 0 |
| Flint | Cold | | Hybrid | 243 | 108 | 2.25 | 1.526 | 1.817 | 0.46 | 0.56±0.04 | 0 |
| Flint | Control | | Hybrid | 243 | 14 | 17.36 | 31.597 | 22.372 | 0.59 | 0.53±0.05 | 0 |
|  | | ΦPSII^h^: 126 SNPs | | | | | | | | | |
| Dent | Cold | | *Per se* | 295 | 256 | 1.15 | 9122 | 10378 | 0.47 | 0.76±0.02 | 23 |
| Dent | Control | | *Per se* | 296 | 129 | 2.30 | 127.3 | 3939.4 | 0.03 | 0.69±0.04 | 100 |
| Flint | Cold | | *Per se* | 241 | 241 | 1 | 9924 | 7719 | 0.56 | 0.64±0.04 | 2 |
| Flint | Control | | *Per se* | 244 | 159 | 1.53 | 1783 | 474 | 0.79 | 0.60±0.05 | 0 |
| Dent | Cold | | Hybrid | 304 | 124 | 2.45 | 0.002 | 0.011 | 0.18 | 0.27±0.07 | 1 |
| Dent | Control | | Hybrid | 304 | 20 | 15.2 | 0.000015 | 0.000042 | 0.27 | 0.45±0.07 | 0 |
| Flint | Cold | | Hybrid | 243 | 23 | 10.57 | 0.015 | 0.012 | 0.56 | 0.32±0.07 | 0 |
| Flint | Control | | Hybrid | 243 | 228 | 1.07 | 0.000009 | 0.0000027 | 0.25 | 0.19±0.12 | 1 |
|  | | Early vigor^i^: 2 SNPs | | | | | | | | | |
| Dent | Cold | | *Per se* | 295 | 136 | 2.17 | 0.131 | 0.395 | 0.25 | 0.51±0.05 | 0 |
| Dent | Control | | *Per se* | 294 | 91 | 3.23 | 0.015 | 0.231 | 0.06 | 0.55±0.04 | 0 |
| Flint | Cold | | *Per se* | 242 | 242 | 1 | 0.133 | 0.241 | 0.35 | 0.37±0.06 | 1 |
| Flint | Control | | *Per se* | 244 | 176 | 1.39 | 0.217 | 0.142 | 0.60 | 0.62±0.04 | 1 |
|  | | Early dry weight: 1 SNPs | | | | | | | | | |
| Dent | Cold | | Hybrid | 304 | 201 | 1.44 | 0.00011 | 0.00086 | 0.11 | 0.47±0.05 | 0 |
| Dent | Control | | Hybrid | 304 | 304 | 1 | 0.00012 | 0.00075 | 0.14 | 0.49±0.06 | 1 |
| Flint | Cold | | Hybrid | 243 | 172 | 1.41 | 0.00071 | 0.00097 | 0.04 | 0.63±0.04 | 0 |
| Flint | Control | | Hybrid | 243 | 129 | 1.88 | 0.00038 | 0.00111 | 0.25 | 0.75±0.03 | 0 |

^a^ n: Total number of inbred lines included in the analysis

^b^ s: Number of groups clustered based on kinship among inbred lines determined by the compression option.

^c^ Compression level is the average number of inbred lines per group estimated as n/s.

d
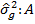
Additive background genetic variance component estimated in Tassel by fitting the K matrix in the MLM without any SNP marker effects.

e
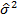
: Residual genotypic variance component estimated in Tassel.

f
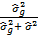
: Proportion of phenotypic variance explained by the K matrix, estimated as background genetic variance divided by total phenotypic variance.

^g^ Relative chlorophyll content.

^h^ Quantum efficiency of PSII.

^i^ Early vigor: subjective score from 1 = weak plants to 9 = vigorous plants.

*h* ^2^  heritability on a line mean-basis

se standard error
