# Supplementary material for: Association mapping for cold tolerance in two large maize inbred panels
Source: BMC Plant Biol. 2016 Jun 6;16:127. doi: 10.1186/s12870-016-0816-2 (PMC4895824; doi:10.1186/s12870-016-0816-2)
Supplement: Additional file 2: Table S2. — SNPs declared as significantly associated to cold tolerance-related traits, chromosome location, P-Value, allele effect estimates, proportion of total variance explained by the SNPs from the association analyses for cold tolerance traits in two association panels of maize inbred lines evaluated in test-crosses under cold and control conditions and per se under control conditions. (DOCX 47 kb) [file 12870_2016_816_MOESM2_ESM.docx]

| Table S2. Significantly SNPs associated to cold tolerance-related traits from the association analyses for cold tolerance. Association panels of maize inbred lines were evaluated in test-crosses under cold and control conditions and *per se* under control conditions. | | | | | | | |
| --- | --- | --- | --- | --- | --- | --- | --- |
| Days to emergence | | | | | | | |
| Trial | Environ. | Genotype | Chromosome | Location | Site | SNP | *P*-Value |
| Dent | Cold | Hybrids | 3 | 202768746 | 18094 | PZE-103148799 | 7.63E-06 |
| Dent | Control | Hybrids | 10 | 50673176 | 46678 | PZE_110032333 | 3.87E-06 |
| Dent | Control | Hybrids | 4 | 45849607 | 20196 | PZE_104035620 | 4.01E-06 |
| Dent | Control | Hybrids | 5 | 24720271 | 25248 | PZE_105039693 | 5.77E-06 |
| Dent | Control | Hybrids | 7 | 16997943 | 34171 | PZE_107018762 | 8.56E-06 |
| Dent | Control | Hybrids | 4 | 124931464 | 21493 | PZE_104063163 | 1.22E-05 |
| Dent | Control | Inbreds | 10 | 113515311 | 48057 | PZE_110059362 | 1.15E-10 |
| Dent | Control | Inbreds | 7 | 133379745 | 36486 | SYN32293 | 2.55E-10 |
| Dent | Control | Inbreds | 1 | 15475258 | 520 | PZE_101025939 | 5.65E-08 |
| Dent | Control | Inbreds | 10 | 83594630 | 47403 | PZE_110043783 | 5.72E-08 |
| Dent | Control | Inbreds | 2 | 201680242 | 12584 | PZB01261.1 | 1.44E-07 |
| Dent | Control | Inbreds | 1 | 224100648 | 5543 | PHM12693.8 | 8.21E-07 |
| Dent | Control | Inbreds | 8 | 308178 | 37804 | PZE_108000369 | 8.95E-07 |
| Dent | Control | Inbreds | 3 | 50102442 | 14729 | PZE_103047484 | 9.30E-07 |
| Dent | Control | Inbreds | 10 | 78869986 | 47294 | PZE_110041280 | 1.48E-06 |
| Dent | Control | Inbreds | 8 | 1371873 | 37834 | SYN4028 | 1.56E-06 |
| Dent | Control | Inbreds | 2 | 16351358 | 8440 | SYN8397 | 5.52E-06 |
| Dent | Control | Inbreds | 5 | 18452570 | 25061 | PUT_163a_93283750_4850 | 6.86E-06 |
| Dent | Control | Inbreds | 2 | 16124292 | 8436 | SYN268 | 6.96E-06 |
| Dent | Control | Inbreds | 1 | 224070120 | 5533 | PZE_101179560 | 7.77E-06 |
| Dent | Control | Inbreds |  |  | 49400 | SYN34975 | 8.41E-06 |
| Dent | Control | Inbreds | 2 | 16122916 | 8435 | SYN266 | 8.86E-06 |
| Dent | Control | Inbreds | 2 | 20196022 | 8553 | ZM011131_0184 | 8.89E-06 |
| Dent | Control | Inbreds | 2 | 16547063 | 8443 | PZE_102034918 | 9.27E-06 |
| Dent | Control | Inbreds | 3 | 97207501 | 15517 | PZE_103064301 | 1.19E-05 |
| Dent | Control | Inbreds | 2 | 220495698 | 13065 | SYN28050 | 1.40E-05 |
| Dent | Control | Inbreds | 10 | 69009147 | 47057 | PZE_110036167 | 9.6×10^-15^ |
| Flint | Cold | Hybrids | 1 | 41854434 | 1282 | SYN29863 | 1.76E-10 |
| Flint | Cold | Hybrids | 1 | 176870110 | 4253 | PZE_101136735 | 1.87E-06 |
| Flint | Cold | Hybrids | 1 | 198232235 | 4797 | SYN28790 | 1.20E-06 |
| Flint | Cold | Hybrids | 1 | 208799094 | 5084 | PZE_101165741 | 1.02E-06 |
| Flint | Cold | Hybrids | 2 | 214215676 | 12964 | PZE_102171003 | 1.18E-05 |
| Flint | Cold | Hybrids | 2 | 229298876 | 13244 | PZE_102185564 | 3.62E-07 |
| Flint | Cold | Hybrids | 2 | 230520732 | 13270 | SYN39027 | 9.84E-11 |
| Flint | Cold | Hybrids | 3 | 48932455 | 14698 | PZE_103046590 | 1.38E-06 |
| Flint | Cold | Hybrids | 3 | 53675536 | 14801 | PZE_103049396 | 1.84E-09 |
| Flint | Cold | Hybrids | 3 | 175965506 | 17281 | PZE_103116489 | 1.83E-10 |
| Flint | Cold | Hybrids | 3 | 229382265 | 18903 | SYN4868 | 1.10E-10 |
| Flint | Cold | Hybrids | 4 | 14125837 | 19375 | SYN8515 | 2.09E-06 |
| Flint | Cold | Hybrids | 4 | 123705446 | 21461 | PZE_104062536 | 1.86E-10 |
| Flint | Cold | Hybrids | 4 | 155228317 | 22147 | SYN4386 | 4.33E-06 |
| Flint | Cold | Hybrids | 6 | 113274586 | 31928 | PZE_106062506 | 2.15E-08 |
| Flint | Cold | Hybrids | 7 | 4238741 | 33829 | PZA02983.28 | 1.19E-05 |
| Flint | Cold | Hybrids | 8 | 2901121 | 37878 | PZE_108002818 | 1.04E-07 |
| Flint | Cold | Hybrids | 8 | 3298822 | 37891 | SYN17933 | 1.66E-10 |
| Flint | Cold | Hybrids | 8 | 7748559 | 38006 | SYN20700 | 1.68E-10 |
| Flint | Cold | Hybrids | 8 | 7760523 | 38011 | SYN20702 | 1.81E-10 |
| Flint | Cold | Hybrids | 8 | 7798037 | 38014 | SYN20710 | 1.39E-10 |
| Flint | Cold | Hybrids | 8 | 8016735 | 38019 | PZE_108007795 | 9.51E-11 |
| Flint | Cold | Hybrids | 8 | 8123084 | 38026 | SYN22840 | 1.54E-10 |
| Flint | Cold | Hybrids | 8 | 82060785 | 39541 | PZE_108048408 | 1.65E-10 |
| Flint | Cold | Hybrids | 8 | 82379213 | 39546 | SYN12845 | 1.87E-10 |
| Flint | Cold | Hybrids | 8 | 82379224 | 39547 | SYN12844 | 7.73E-06 |
| Flint | Cold | Hybrids | 8 | 82384876 | 39548 | SYN12841 | 7.73E-06 |
| Flint | Cold | Hybrids | 8 | 83734213 | 39573 | PZE_108048978 | 1.48E-10 |
| Flint | Cold | Hybrids | 8 | 84339799 | 39589 | PZE_108049207 | 1.81E-10 |
| Flint | Cold | Hybrids | 8 | 84607800 | 39593 | PZE_108049268 | 1.81E-10 |
| Flint | Cold | Hybrids | 8 | 84612625 | 39595 | PZE_108049272 | 1.81E-10 |
| Flint | Cold | Hybrids | 8 | 84697712 | 39596 | PZE_108049289 | 1.81E-10 |
| Flint | Cold | Hybrids | 8 | 84724644 | 39597 | PZE_108049296 | 1.81E-10 |
| Flint | Cold | Hybrids | 8 | 84786202 | 39600 | PZE_108049314 | 1.76E-10 |
| Flint | Cold | Hybrids | 8 | 84986404 | 39608 | PZE_108049376 | 1.76E-10 |
| Flint | Cold | Hybrids | 8 | 85123531 | 39612 | PZE_108049452 | 1.56E-10 |
| Flint | Cold | Hybrids | 8 | 85312502 | 39617 | PZE_108049544 | 4.31E-06 |
| Flint | Cold | Hybrids | 8 | 85786934 | 39622 | PZE_108049609 | 1.43E-10 |
| Flint | Cold | Hybrids | 8 | 86029780 | 39632 | PZE_108049707 | 1.82E-10 |
| Flint | Cold | Hybrids | 8 | 86029983 | 39633 | PZE_108049714 | 1.43E-06 |
| Flint | Cold | Hybrids | 8 | 86403100 | 39635 | PZE_108049805 | 1.86E-10 |
| Flint | Cold | Hybrids | 8 | 86585570 | 39638 | PZE_108050204 | 1.40E-06 |
| Flint | Cold | Hybrids | 8 | 86959998 | 39646 | PZE_108050089 | 1.21E-05 |
| Flint | Cold | Hybrids | 8 | 87033018 | 39649 | SYN36274 | 1.08E-06 |
| Flint | Cold | Hybrids | 8 | 88275629 | 39675 | PZE_108050575 | 1.79E-10 |
| Flint | Cold | Hybrids | 8 | 88439739 | 39678 | PZE_108050624 | 1.83E-10 |
| Flint | Cold | Hybrids | 8 | 88813606 | 39683 | PZE_108050769 | 1.54E-07 |
| Flint | Cold | Hybrids | 8 | 88813911 | 39684 | PZE_108050778 | 1.54E-07 |
| Flint | Cold | Hybrids | 8 | 88889302 | 39685 | PZE_108050819 | 1.54E-07 |
| Flint | Cold | Hybrids | 8 | 88893085 | 39686 | PZE_108050812 | 1.70E-10 |
| Flint | Cold | Hybrids | 8 | 90738162 | 39729 | SYN30907 | 1.06E-05 |
| Flint | Cold | Hybrids | 8 | 90966409 | 39738 | SYN23112 | 1.39E-05 |
| Flint | Cold | Hybrids | 8 | 91178679 | 39742 | PZE_108051664 | 6.34E-06 |
| Flint | Cold | Hybrids | 8 | 94302320 | 39800 | PZE_108052858 | 4.43E-06 |
| Flint | Cold | Hybrids | 8 | 94592195 | 39808 | PZE_108052970 | 1.82E-10 |
| Flint | Cold | Hybrids | 8 | 95127963 | 39827 | PZE_108053433 | 1.86E-10 |
| Flint | Cold | Hybrids | 8 | 95127991 | 39828 | PZE_108053434 | 2.12E-06 |
| Flint | Cold | Hybrids | 8 | 95631921 | 39835 | PZE_108053608 | 5.55E-06 |
| Flint | Cold | Hybrids | 8 | 95715685 | 39839 | PZE_108053677 | 9.63E-07 |
| Flint | Cold | Hybrids | 8 | 95745862 | 39843 | PZE_108053763 | 1.12E-05 |
| Flint | Cold | Hybrids | 8 | 96076158 | 39854 | SYN3403 | 3.76E-06 |
| Flint | Cold | Hybrids | 8 | 96270536 | 39857 | PZE_108054157 | 2.22E-06 |
| Flint | Cold | Hybrids | 8 | 96271172 | 39858 | PZE_108054165 | 6.71E-08 |
| Flint | Cold | Hybrids | 8 | 96326653 | 39859 | PZE_108054215 | 3.10E-07 |
| Flint | Cold | Hybrids | 8 | 96832502 | 39862 | PZE_108054420 | 1.05E-06 |
| Flint | Cold | Hybrids | 8 | 97028659 | 39863 | PZE_108054494 | 3.58E-11 |
| Flint | Cold | Hybrids | 8 | 97669049 | 39870 | PZE_108054728 | 1.75E-10 |
| Flint | Cold | Hybrids | 8 | 99161510 | 39887 | PZE_108055186 | 3.09E-06 |
| Flint | Cold | Hybrids | 8 | 99201341 | 39893 | PZE_108055245 | 1.51E-10 |
| Flint | Cold | Hybrids | 8 | 101209781 | 39931 | SYN17876 | 3.49E-06 |
| Flint | Cold | Hybrids | 8 | 101267152 | 39934 | PZE_108056415 | 5.04E-06 |
| Flint | Cold | Hybrids | 8 | 101267183 | 39935 | PZE_108056416 | 1.85E-10 |
| Flint | Cold | Hybrids | 8 | 131799613 | 40753 | SYN37265 | 1.31E-05 |
| Flint | Cold | Hybrids | 9 | 127450684 | 44734 | PZE_109079376 | 1.06E-10 |
| Flint | Cold | Hybrids | 9 | 136619095 | 44985 | PZE_109088195 | 1.23E-05 |
| Flint | Cold | Hybrids | 10 | 98368399 | 47744 | PZE_110052085 | 1.93E-08 |
| Flint | Cold | Hybrids | 10 | 111416827 | 48007 | PZE_110057940 | 1.38E-09 |
| Flint | Control | Hybrids | 6 | 152459743 | 33037 | PZE_106098900 | 1.78E-08 |
| Flint | Control | Hybrids | 2 | 17477443 | 8461 | SYN18095 | 2.12E-08 |
| Flint | Control | Hybrids | 6 | 134098356 | 32454 | PZE_106078534 | 2.84E-07 |
| Flint | Control | Hybrids | 9 | 36297112 | 42930 | PZE_109031375 | 3.45E-07 |
| Flint | Control | Hybrids | 4 | 40810953 | 20072 | PZE_104033114 | 3.48E-07 |
| Flint | Control | Hybrids | 3 | 108235945 | 15653 | PZE_103061052 | 4.29E-07 |
| Flint | Control | Hybrids | 9 | 69868619 | 43498 | PZE_109042675 | 5.46E-07 |
| Flint | Control | Hybrids | 8 | 52902989 | 38910 | PZE_108035671 | 5.68E-07 |
| Flint | Control | Hybrids | 9 | 110714972 | 44352 | PZE_109066885 | 5.71E-07 |
| Flint | Control | Hybrids | 6 | 130964172 | 32359 | SYN27645 | 6.51E-07 |
| Flint | Control | Hybrids | 7 | 21188966 | 34287 | PZE_107021672 | 6.67E-07 |
| Flint | Control | Hybrids | 1 | 237735668 | 5897 | PZE_101191494 | 7.56E-07 |
| Flint | Control | Hybrids | 4 | 1222288 | 19052 | PZE_104005416 | 8.04E-07 |
| Flint | Control | Hybrids | 8 | 109835767 | 40126 | PZE_108061262 | 9.59E-07 |
| Flint | Control | Hybrids | 8 | 54444675 | 38934 | PZE_108036058 | 1.21E-06 |
| Flint | Control | Hybrids | 6 | 130133933 | 32342 | PZE_106075075 | 1.22E-06 |
| Flint | Control | Hybrids | 10 | 140049011 | 48804 | PZE_110090887 | 1.24E-06 |
| Flint | Control | Hybrids | 9 | 36743218 | 42933 | PZE_109031607 | 1.27E-06 |
| Flint | Control | Hybrids | 5 | 178243962 | 28501 | SYN8802 | 2.59E-06 |
| Flint | Control | Hybrids | 2 | 33499030 | 8927 | PZE_102055564 | 4.78E-06 |
| Flint | Control | Hybrids | 1 | 27095647 | 862 | PZE_101039839 | 5.03E-06 |
| Flint | Control | Hybrids | 5 | 209934483 | 29413 | PZE_105166527 | 5.78E-06 |
| Flint | Control | Hybrids | 7 | 112972092 | 35985 | PZE_107058951 | 5.93E-06 |
| Flint | Control | Hybrids | 10 | 87799475 | 47499 | PZE_110046826 | 7.05E-06 |
| Flint | Control | Hybrids | 5 | 176909050 | 28465 | PZE_105120292 | 7.98E-06 |
| Flint | Control | Inbreds | 9 | 102197031 | 44150 | PZE_109060565 | 6.50E-07 |
| Flint | Control | Inbreds | 9 | 102785317 | 44160 | SYN34033 | 8.25E-07 |
| Flint | Control | Inbreds | 9 | 102786397 | 44161 | PZE_109061001 | 8.25E-07 |
| Flint | Control | Inbreds | 9 | 101827139 | 44142 | PZE_109060192 | 6.08E-06 |
| Flint | Control | Inbreds | 10 | 147154005 | 49054 | SYN22556 | 6.80E-06 |
| Flint | Control | Inbreds | 8 | 68310390 | 39221 | PZE_108041881 | 9.20E-06 |
| Flint | Control | Inbreds | 8 | 71791045 | 39297 | PZE_108043101 | 9.26E-06 |
| Flint | Control | Inbreds | 6 | 162373180 | 33425 | SYN24414 | 9.36E-06 |
| Flint | Control | Inbreds | 8 | 51743919 | 38893 | PZE_108035422 | 9.99E-06 |
| Flint | Control | Inbreds | 8 | 48874246 | 38870 | PZE_108035025 | 1.11E-05 |
| Flint | Control | Inbreds | 8 | 98655047 | 39877 | PZE_108055040 | 1.14E-05 |
| ΦPSII | | | | | | | |
| Dent | Control | Hybrids | 10 | 62997257 | 46924 | PZE_110033415 | 7.34E-06 |
| Dent | Control | Inbreds | 10 | 69009147 | 47057 | PZE_110036167 | 3.21E-12 |
| Dent | Control | Inbreds | 9 | 5516605 | 42189 | PZE_109005097 | 4.00E-09 |
| Dent | Control | Inbreds | 7 | 17697607 | 34202 | SYN27394 | 1.02E-08 |
| Dent | Control | Inbreds | 7 | 133379745 | 36486 | SYN32293 | 1.03E-08 |
| Dent | Control | Inbreds | 1 | 15475258 | 520 | PZE_101025939 | 2.28E-08 |
| Dent | Control | Inbreds | 7 | 17480898 | 34192 | SYN15992 | 7.61E-08 |
| Dent | Control | Inbreds | 2 | 18618444 | 8494 | PZE_102038457 | 8.76E-08 |
| Dent | Control | Inbreds | 1 | 224100648 | 5543 | PHM12693.8 | 3.75E-07 |
| Dent | Control | Inbreds | 7 | 17522075 | 34196 | SYN15994 | 6.65E-07 |
| Dent | Control | Inbreds | 5 | 120329460 | 27292 | PZE_105088725 | 7.51E-07 |
| Dent | Control | Inbreds | 3 | 50102442 | 14729 | PZE_103047484 | 7.61E-07 |
| Dent | Control | Inbreds | 10 | 83594630 | 47403 | PZE_110043783 | 1.24E-06 |
| Dent | Control | Inbreds | 7 | 150041861 | 36971 | SYN1092 | 1.27E-06 |
| Dent | Control | Inbreds | 1 | 224070120 | 5533 | PZE_101179560 | 1.89E-06 |
| Dent | Control | Inbreds | 5 | 6358369 | 24709 | SYN38075 | 3.49E-06 |
| Dent | Control | Inbreds | 5 | 60250718 | 26165 | ZM013788_0550 | 4.91E-06 |
| Dent | Control | Inbreds | 10 | 88684441 | 47536 | SYN20534 | 5.44E-06 |
| Dent | Control | Inbreds | 3 | 225447101 | 18777 | PZE_103180492 | 7.05E-06 |
| Dent | Control | Inbreds | 3 | 225453553 | 18780 | SYNGENTA15567 | 8.33E-06 |
| Dent | Control | Inbreds | 5 | 51435922 | 25952 | PZE_105054666 | 9.02E-06 |
| Dent | Control | Inbreds | 2 | 275341 | 7806 | PZE_102000151 | 1.06E-05 |
| Dent | Control | Inbreds | 3 | 225452666 | 18778 | PZE_103180497 | 1.20E-05 |
| Dent | Control | Inbreds | 10 | 78869986 | 47294 | PZE_110041280 | 1.20E-05 |
| Flint | Cold | Hybrids | 4 | 15531421 | 19406 | PZE_104015863 | 7.35E-06 |
| Flint | Control | Inbreds | 3 | 4712666 | 13632 | SYN5647 | 1.42E-06 |
| Flint | Control | Inbreds | 3 | 9067379 | 13739 | SYN13699 | 1.37E-07 |
| Flint | Control | Inbreds | 3 | 9070842 | 13740 | PZE_103015901 | 1.17E-07 |
| Flint | Control | Inbreds | 3 | 9317760 | 13744 | SYN21254 | 3.78E-08 |
| Flint | Control | Inbreds | 3 | 10283017 | 13773 | PZE_103017596 | 1.15E-07 |
| Flint | Control | Inbreds | 3 | 15422865 | 13898 | SYN26604 | 3.21E-08 |
| Flint | Control | Inbreds | 3 | 15434455 | 13903 | PZE_103023322 | 1.13E-07 |
| Flint | Control | Inbreds | 3 | 16309019 | 13916 | PZE_103023739 | 1.40E-07 |
| Flint | Control | Inbreds | 3 | 25215408 | 14133 | SYN6603 | 1.09E-07 |
| Flint | Control | Inbreds | 3 | 25217383 | 14139 | PZE_103032446 | 8.57E-08 |
| Flint | Control | Inbreds | 3 | 25862727 | 14147 | SYN36774 | 1.10E-07 |
| Flint | Control | Inbreds | 3 | 85663321 | 15289 | PZE_103069212 | 4.37E-06 |
| Flint | Control | Inbreds | 3 | 87713637 | 15330 | PZE_103067949 | 7.64E-08 |
| Flint | Control | Inbreds | 3 | 87786034 | 15338 | SYN15223 | 1.38E-07 |
| Flint | Control | Inbreds | 3 | 87803865 | 15339 | PZE_103067856 | 1.40E-07 |
| Flint | Control | Inbreds | 3 | 88375143 | 15345 | PZE_103067736 | 1.40E-07 |
| Flint | Control | Inbreds | 3 | 88388153 | 15347 | PZE_103067710 | 1.34E-07 |
| Flint | Control | Inbreds | 3 | 88388226 | 15348 | PZE_103067709 | 1.19E-07 |
| Flint | Control | Inbreds | 3 | 88389983 | 15350 | PZE_103067702 | 1.38E-07 |
| Flint | Control | Inbreds | 3 | 88474026 | 15353 | PZE_103067657 | 9.46E-08 |
| Flint | Control | Inbreds | 3 | 88923628 | 15365 | PZE_103067394 | 1.33E-07 |
| Flint | Control | Inbreds | 3 | 89418323 | 15368 | PZE_103067232 | 1.36E-07 |
| Flint | Control | Inbreds | 3 | 89432165 | 15373 | PUT_163a_18176670_1449 | 1.42E-07 |
| Flint | Control | Inbreds | 3 | 91359960 | 15405 | SYN28776 | 1.13E-07 |
| Flint | Control | Inbreds | 3 | 89433406 | 15377 | PZE_103067169 | 1.25E-07 |
| Flint | Control | Inbreds | 3 | 91676886 | 15414 | PZE_103066354 | 9.12E-08 |
| Flint | Control | Inbreds | 3 | 92535545 | 15428 | PUT_163a_74245700_3703 | 1.39E-07 |
| Flint | Control | Inbreds | 3 | 92535687 | 15429 | PUT_163a_74245700_3707 | 1.34E-07 |
| Flint | Control | Inbreds | 3 | 92535692 | 15430 | PUT_163a_74245700_3702 | 1.34E-07 |
| Flint | Control | Inbreds | 3 | 92542471 | 15435 | PZE_103065820 | 1.27E-07 |
| Flint | Control | Inbreds | 3 | 94333865 | 15474 | PZE_103065175 | 1.18E-07 |
| Flint | Control | Inbreds | 3 | 94797222 | 15478 | PZE_103065036 | 1.18E-07 |
| Flint | Control | Inbreds | 3 | 95267821 | 15485 | PZE_103064889 | 1.42E-07 |
| Flint | Control | Inbreds | 3 | 95267975 | 15479 | PZE_103065029 | 1.42E-07 |
| Flint | Control | Inbreds | 3 | 95288372 | 15486 | PZE_103064888 | 1.42E-07 |
| Flint | Control | Inbreds | 3 | 95408631 | 15487 | PZE_103064882 | 1.42E-07 |
| Flint | Control | Inbreds | 3 | 95566344 | 15490 | PZE_103064809 | 1.15E-07 |
| Flint | Control | Inbreds | 3 | 95754493 | 15492 | PZE_103064739 | 1.42E-07 |
| Flint | Control | Inbreds | 3 | 96011509 | 15493 | PZE_103064695 | 1.15E-07 |
| Flint | Control | Inbreds | 3 | 96226819 | 15497 | PZE_103064649 | 1.27E-07 |
| Flint | Control | Inbreds | 3 | 96240433 | 15498 | PZE_103064646 | 1.24E-07 |
| Flint | Control | Inbreds | 3 | 96240598 | 15499 | PZE_103064643 | 1.24E-07 |
| Flint | Control | Inbreds | 3 | 96250102 | 15502 | PZE_103064628 | 1.42E-07 |
| Flint | Control | Inbreds | 3 | 96317357 | 15503 | PZE_103064624 | 1.24E-07 |
| Flint | Control | Inbreds | 3 | 96366130 | 15504 | PZE_103064615 | 1.24E-07 |
| Flint | Control | Inbreds | 3 | 96706581 | 15509 | PZE_103064491 | 1.42E-07 |
| Flint | Control | Inbreds | 3 | 107571143 | 15640 | PZE_103061205 | 1.33E-07 |
| Flint | Control | Inbreds | 3 | 107571442 | 15641 | SYN23898 | 1.30E-07 |
| Flint | Control | Inbreds | 3 | 107785354 | 15645 | PZE_103061139 | 1.26E-07 |
| Flint | Control | Inbreds | 3 | 108707871 | 15670 | PUT_163a_149079952_852 | 1.19E-07 |
| Flint | Control | Inbreds | 3 | 110461222 | 15689 | PZE_103060293 | 1.10E-07 |
| Flint | Control | Inbreds | 3 | 110670793 | 15697 | PZE_103060189 | 1.35E-07 |
| Flint | Control | Inbreds | 3 | 111058595 | 15700 | PZE_103060044 | 1.35E-07 |
| Flint | Control | Inbreds | 3 | 111185343 | 15701 | PZE_103060008 | 1.35E-07 |
| Flint | Control | Inbreds | 3 | 111328408 | 15707 | PZE_103059944 | 1.36E-07 |
| Flint | Control | Inbreds | 3 | 111347232 | 15709 | SYN31954 | 1.35E-07 |
| Flint | Control | Inbreds | 3 | 111842810 | 15720 | SYN4095 | 1.35E-07 |
| Flint | Control | Inbreds | 3 | 112044687 | 15724 | PZE_103059709 | 1.42E-07 |
| Flint | Control | Inbreds | 4 | 5010683 | 19185 | SYNGENTA2238 | 1.26E-05 |
| Flint | Control | Inbreds | 4 | 41663874 | 20082 | PZE_104033329 | 1.34E-07 |
| Flint | Control | Inbreds | 4 | 165639599 | 22387 | SYN2280 | 1.23E-07 |
| Flint | Control | Inbreds | 4 | 165640912 | 22388 | PZE_104089648 | 1.13E-07 |
| Flint | Control | Inbreds | 4 | 165658268 | 22391 | PZE_104089682 | 6.89E-08 |
| Flint | Control | Inbreds | 4 | 165659907 | 22392 | PZE_104089684 | 9.15E-08 |
| Flint | Control | Inbreds | 4 | 165666018 | 22396 | PUT_163a_31405788_1909 | 9.85E-08 |
| Flint | Control | Inbreds | 4 | 172833619 | 22578 | SYN2271 | 5.39E-08 |
| Flint | Control | Inbreds | 4 | 173210826 | 22587 | SYN2244 | 6.19E-08 |
| Flint | Control | Inbreds | 4 | 173310967 | 22590 | PZE_104096880 | 1.22E-07 |
| Flint | Control | Inbreds | 4 | 173331938 | 22592 | SYN2088 | 1.35E-07 |
| Flint | Control | Inbreds | 4 | 175379156 | 22648 | PZE_104099048 | 8.65E-06 |
| Flint | Control | Inbreds | 4 | 175735834 | 22654 | PZE_104099233 | 1.42E-07 |
| Flint | Control | Inbreds | 4 | 176476512 | 22669 | PZE_104099606 | 1.40E-07 |
| Flint | Control | Inbreds | 4 | 177055653 | 22684 | SYN2327 | 6.29E-06 |
| Flint | Control | Inbreds | 4 | 179619049 | 22753 | PZE_104103128 | 5.90E-08 |
| Flint | Control | Inbreds | 4 | 211481615 | 23539 | PZE_104130586 | 2.99E-06 |
| Flint | Control | Inbreds | 4 | 212042926 | 23541 | PZE_104130779 | 1.41E-07 |
| Flint | Control | Inbreds | 4 | 212042949 | 23542 | PZE_104130780 | 1.40E-07 |
| Flint | Control | Inbreds | 4 | 212043051 | 23543 | PZE_104130783 | 1.41E-07 |
| Flint | Control | Inbreds | 4 | 215234701 | 23620 | SYN17464 | 5.53E-06 |
| Flint | Control | Inbreds | 4 | 219147127 | 23706 | PZE_104134776 | 8.08E-08 |
| Flint | Control | Inbreds | 4 | 223107502 | 23806 | SYN37142 | 7.78E-08 |
| Flint | Control | Inbreds | 4 | 224037126 | 23827 | SYN8646 | 1.31E-07 |
| Flint | Control | Inbreds | 4 | 224730780 | 23841 | SYN22433 | 1.08E-07 |
| Flint | Control | Inbreds | 4 | 226268349 | 23885 | SYN30108 | 1.48E-08 |
| Flint | Control | Inbreds | 4 | 226941361 | 23900 | SYN34780 | 9.91E-08 |
| Flint | Control | Inbreds | 4 | 226941441 | 23901 | SYN34782 | 1.14E-07 |
| Flint | Control | Inbreds | 4 | 227209639 | 23909 | PZE_104139552 | 5.12E-08 |
| Flint | Control | Inbreds | 4 | 227209956 | 23910 | PZE_104139559 | 1.29E-07 |
| Flint | Control | Inbreds | 4 | 227214814 | 23913 | PZE_104139569 | 1.24E-07 |
| Flint | Control | Inbreds | 4 | 228033802 | 23936 | PZE_104140021 | 5.18E-08 |
| Flint | Control | Inbreds | 5 | 181584459 | 28591 | PZE_105124576 | 1.35E-07 |
| Flint | Control | Inbreds | 5 | 181586455 | 28592 | PZE_105124586 | 1.28E-07 |
| Flint | Control | Inbreds | 5 | 181889242 | 28610 | SYN20651 | 1.42E-07 |
| Flint | Control | Inbreds | 5 | 182449952 | 28625 | PZE_105125294 | 1.41E-07 |
| Flint | Control | Inbreds | 5 | 182456293 | 28628 | SYN15130 | 1.14E-07 |
| Flint | Control | Inbreds | 5 | 182459447 | 28629 | SYN15131 | 1.41E-07 |
| Flint | Control | Inbreds | 9 | 109124656 | 44316 | PZE_109065991 | 1.00E-05 |
| Flint | Control | Inbreds | 9 | 123637645 | 44650 | PZE_109076475 | 8.09E-08 |
| Flint | Control | Inbreds | 10 | 5873622 | 45800 | SYN16720 | 1.18E-07 |
| Flint | Control | Inbreds |  |  | 49517 | PZB01689.2 | 1.06E-07 |
| Early vigor | | | | | | | |
| Flint | Control | Hybrids | 9 | 134569704 | 44931 | PZE_109085971 | 1.27E-05 |
