# Supplementary material for: Association mapping for cold tolerance in two large maize inbred panels
Source: BMC Plant Biol. 2016 Jun 6;16:127. doi: 10.1186/s12870-016-0816-2 (PMC4895824; doi:10.1186/s12870-016-0816-2)
Supplement: Additional file 3: Table S3. — Candidate genes from the association analyses for cold tolerance traits in two association panels of maize. Genes in a 1400 kb interval surrounding the significant SNPs are listed with their identifier. The gene closest to the significant SNP is indicated in bold. (DOCX 34 kb) [file 12870_2016_816_MOESM3_ESM.docx]

| Table S3. Candidate genes from the association analyses for cold tolerance traits in two association panels of maize. Genes in an 1400 kb interval surrounding the significant SNPs are listed with their identifier. The gene closest to the significant SNP is indicated in bold. | | | | | |
| --- | --- | --- | --- | --- | --- |
| Chromosome | Position (pb) | Gene | Gene Start (pb) | Gene End (pb) | Function / Subcellular localization |
| 3 | 145737736 | GRMZM2G061305 | 144595369 | 145596953 | Transposon protein / ? |
|  |  | GRMZM2G122431 | 145209829 | 145213170 | cullulose biosynthetic process / membrane |
|  |  | GRMZM2G113096 | 145216388 | 145218746 | translation initiation factor activity / cytoplasm |
|  |  | AC233904.1_FG002 | 145247001 | 145253166 | ? / ? |
|  |  | GRMZM2G097972 | 145306659 | 145307318 | ? / ? |
|  |  | GRMZM2G174479 | 145397996 | 145399691 | ? / ? |
|  |  | GRMZM2G097182 | 145467972 | 145468863 | transcription factor activity / nucleus |
|  |  | GRMZM2G097258 | 145470464 | 145473438 | AMP dimethylallyltransferase activity / cytosol |
|  |  | GRMZM2G397948 | 145506612 | 145514316 | ? / ? |
|  |  | GRMZM2G097316 | 145514689 | 145516008 | Root cap protein / ? |
|  |  | GRMZM2G097340 | 145521907 | 145523405 | ? / ? |
|  |  | GRMZM2G061206 | 145636198 | 145639549 | Antiporter |
|  |  | GRMZM2G061127 | 145642310 | 145643935 | Proteolysis / Chloroplast stroma localization |
|  |  | **GRMZM2G174274** | **145762729** | **145764119** | **Signal transduction / Intracellular** |
|  |  | GRMZM2G174249 | 145763792 | 145766257 | Carbohydrate, response to wounding / Cell wall vacuole |
|  |  | GRMZM2G174221 | 145771397 | 145774255 | Vacuole |
|  |  | GRMZM2G174196 | 145774369 | 145777597 | Protein binding |
|  |  | GRMZM2G174137 | 145786387 | 145795884 | Catalytic activity |
|  |  | GRMZM2G074241 | 145796992 | 145799980 | ATPase activity / Membrane |
|  |  | GRMZM2G375807 | 145817237 | 145825052 | ATPase activity / Membrane |
|  |  | GRMZM2G071270 | 145834548 | 145844492 | Superoxido dismutase activity / ? |
|  |  | GRMZM2G371912 | 145855491 | 145858353 | N-acetyltransferase activity / ? |
|  |  | GRMZM5G832423 | 145927044 | 145928334 | ? / ? |
|  |  | GRMZM2G139462 | 145931252 | 145934946 | UMP kinase activity / chloroplast |
|  |  | GRMZM2G047457 | 146060122 | 146061766 | iron ion transport / chloroplast envelope |
|  |  | GRMZM2G047409 | 146065990 | 146066819 | iron ion transport / ? |
|  |  | GRMZM2G344799 | 146068653 | 146073118 | ? / ? |
|  |  | GRMZM2G071433 | 146123771 | 146124958 | ? / ? |
|  |  | GRMZM2G309512 | 146284125 | 146287100 | protein serine/threonine kinase activity / ? |
|  |  | GRMZM2G309479 | 146286651 | 146287229 | ? / ? |
| 1 | 201477347 | GRMZM5G829831 | 200718342 | 200730230 | ? / ? |
|  |  | GRMZM2G152156 | 200749036 | 200755504 | ? / ? |
|  |  | GRMZM2G133413 | 200759099 | 200762378 | ? / nucleus |
|  |  | GRMZM2G157772 | 200865263 | 200867080 | catalytic activity / ? |
|  |  | GRMZM2G346920 | 200897499 | 200899336 | ZF-HD protein dimerisation region containing protein / ? |
|  |  | GRMZM2G004191 | 201205861 | 201208156 | ? / ? |
|  |  | GRMZM2G004079 | 201226717 | 201229957 | protein serine/threonine kinase activity / ? |
|  |  | GRMZM2G419024 | 201439240 | 201443106 | Triosephosphate isomerase activity |
|  |  | **GRMZM5G899800** | **201502394** | **201505169** | **Protein heterodimerization activity** |
|  |  | GRMZM2G416069 | 201513870 | 201519179 | Protein heterodimerization activity / Cohesin complex |
|  |  | GRMZM2G115730 | 201519254 | 201520692 | Unknown |
|  |  | GRMZM2G115750 | 201522268 | 201525873 | Receptor / Membrane, ER, Golgi |
|  |  | GRMZM2G017045 | 201661039 | 201662079 | electron carrier activity / Thylakoid membrane |
|  |  | GRMZM2G318803 | 201662604 | 201667147 | histone-lysine N-methyltransferase activity / nucleus |
|  |  | GRMZM2G318794 | 201668883 | 201669657 | Protein transport / mitochondria |
|  |  | GRMZM2G016210 | 201673513 | 201678322 | ? / ? |
|  |  | GRMZM2G148896 | 201771782 | 201775619 | ? / ? |
|  |  | GRMZM2G148867 | 201775585 | 201782559 | protein disulfide oxidoreductase activity / ? |
|  |  | GRMZM2G464469 | 201881282 | 201882124 | ? / ? |
|  |  | GRMZM2G165011 | 201882383 | 201884831 | histone-lysine N-methyltransferase activity / nucleus |
|  |  | GRMZM2G056686 | 201904943 | 201909743 | 7-hydroxymethyl chlorophyll a reductase activity / ? |
|  |  | GRMZM2G028386 | 201993810 | 201995393 | transcription factor activity / nucleus |
|  |  | GRMZM2G493946 | 202004350 | 202007593 | ? / ? |
|  |  | GRMZM5G852533 | 202013191 | 202018034 | transcription cofactor activity / ? |
|  |  | GRMZM5G833477 | 202028504 | 202031821 | ? / ? |
|  |  | GRMZM2G164872 | 202032231 | 202033811 | ? / ? |
| 4 | 172689894 | GRMZM2G702697 | 171830828 | 171831597 | ? / ? |
|  |  | AC189280.3_FG001 | 171871760 | 171875450 | protein binding / ? |
|  |  | GRMZM2G466532 | 171938025 | 171939815 | ? / ? |
|  |  | GRMZM2G466545 | 171946555 | 171952268 | ? / membrane |
|  |  | GRMZM2G164854 | 171951352 | 171955233 | protein binding / ? |
|  |  | GRMZM2G034005 | 172039830 | 172048490 | protein binding / plasma membrane |
|  |  | GRMZM2G062084 | 172084974 | 172089808 | microtubule binding / microtubule |
|  |  | GRMZM2G075336 | 172109718 | 172113857 | nucleoside-triphosphatase activity / ? |
|  |  | GRMZM2G075124 | 172114902 | 172119147 | protein serine/threonine/tyrosine kinase activity / nucleus |
|  |  | GRMZM2G023899 | 172138534 | 172139924 | ? / ? |
|  |  | GRMZM2G140901 | 172222465 | 172224718 | DNA binding / nucleus |
|  |  | GRMZM2G107109 | 172286685 | 172295419 | ? / nucleus |
|  |  | GRMZM2G140524 | 172338861 | 172347926 | ? / nucleus |
|  |  | GRMZM2G171163 | 172386710 | 172389864 | ? / ? |
|  |  | GRMZM2G027075 | 172459736 | 172468782 | ? / nucleus |
|  |  | GRMZM2G027302 | 172473806 | 172475786 | ? / ? |
|  |  | GRMZM2G364068 | 172551715 | 172560136 | nucleic acid binding / ? |
|  |  | **GRMZM2G130043** | **172635729** | **172706662** | **Starch synthase** |
|  |  | GRMZM2G130002 | 172714527 | 172719310 | Protein binding / Plasma membrane |
|  |  | GRMZM2G129979 | 172721820 | 172724795 | G10 protein / Nucleous |
|  |  | GRMZM2G178398 | 172777639 | 172783737 | Epsin like protein |
|  |  | GRMZM2G379005 | 172789633 | 172794795 | transcription factor activity / ? |
|  |  | AC186156.3_FG005 | 172823746 | 172824357 | ? / ? |
|  |  | AC186156.3_FG004 | 172851417 | 172852028 | ? / ? |
|  |  | GRMZM2G162007 | 172864338 | 172866690 | oxidoreductase activity / ? |
|  |  | GRMZM2G108364 | 173022083 | 173025239 | 3'(2'),5'-bisphosphate nucleotidase activity / ? |
|  |  | GRMZM2G410487 | 173025671 | 173026879 | protein binding / ? |
|  |  | GRMZM2G108284 | 173027519 | 173031230 | ? / ? |
|  |  | GRMZM2G108712 | 173062872 | 173064850 | DNA polymerase processivity factor activity / nucleolus |
|  |  | GRMZM2G054007 | 173206648 | 173207145 | ? / ? |
|  |  | GRMZM2G054012 | 173207225 | 173209196 | structural constituent of ribosome / nucleolus |
|  |  | GRMZM2G054065 | 173210750 | 173211582 | ? / chloroplast |
|  |  | GRMZM2G054076 | 173210996 | 173223753 | heat shock protein binding / thylakoid membrane |
|  |  | GRMZM2G054210 | 173239464 | 173253984 | protein transporter activity / golgi apparatus |
|  |  | GRMZM5G817422 | 173288239 | 173290211 | ? / ? |
|  |  | GRMZM2G178955 | 173337451 | 173342631 | phospholipid binding / clathrin-coated vesicle |
|  |  | GRMZM2G178919 | 173344237 | 173347777 | ? / ? |
|  |  | GRMZM5G806771 | 173359568 | 173362218 | structural constituent of ribosome / ribosome |
| 1 | 73380804 | AC191589.3_FG003 | 72606681 | 72607465 | ? / ? |
|  |  | GRMZM2G137502 | 72625013 | 72627421 | regulation of transcription / ? |
|  |  | AC177908.3_FG003 | 72698780 | 72699511 | ? / ? |
|  |  | GRMZM2G096682 | 72710256 | 72713376 | amino acid binding / ? |
|  |  | GRMZM5G866989 | 72731051 | 72732328 | ? / ? |
|  |  | AC177908.3_FG006 | 72747838 | 72748728 | ? / ? |
|  |  | GRMZM2G147301 | 72776823 | 72778247 | ? / ? |
|  |  | AC177908.3_FG002 | 72808429 | 72813891 | pectin biosynthetic process / Golgi membrane |
|  |  | GRMZM2G391705 | 72851014 | 72852064 | ? / ? |
|  |  | GRMZM2G351259 | 72891989 | 72893894 | oxidation-reduction process / ? |
|  |  | AC212231.3_FG003 | 73108916 | 73110754 | ? / ? |
|  |  | AC212231.3_FG002 | 73129231 | 73130374 | ? / ? |
|  |  | GRMZM2G117930 | 73239931 | 73240968 | ? / ? |
|  |  | GRMZM2G074946 | 73254414 | 73259651 | carbohydrate metabolic process / ? |
|  |  | **GRMZM2G470442 (GE2)** | **73382596** | **73385374** | **Oxidoreductase activity (monooxygenase) / ?** |
|  |  | GRMZM2G172244 | 73387395 | 73390846 | Unknown |
|  |  | GRMZM2G067371 | 73517191 | 73520282 | Signal transduction / ? |
|  |  | GRMZM2G074102 | 73882293 | 73888903 | ? / nucleolus, plasma membrane, endoplasmic reticulum |
|  |  | GRMZM2G074208 | 73889513 | 73894048 | methyltransferase activity / ? |
|  |  | GRMZM5G849164 | 73923244 | 73923976 | ? / ? |
|  |  | GRMZM2G125241 | 73974267 | 73976401 | Glucan endo-1,3-beta-glucosidase / ? |
|  |  | GRMZM2G517730 | 74004666 | 74005391 | ? / ? |
|  |  | GRMZM2G171022 | 74054142 | 74056560 | protein binding / ? |
| 4 | 20738948 | GRMZM2G309275 | 19868677 | 19877156 | oxidation-reduction process / ? |
|  |  | GRMZM2G098102 | 19936806 | 19938403 | cysteine-type peptidase activity / ? |
|  |  | GRMZM2G351347 | 19967150 | 19968544 | potassium ion transmembrane transport / vacuolar membrane |
|  |  | GRMZM2G166870 | 20001587 | 20003154 | cysteine-type peptidase activity / ? |
|  |  | GRMZM2G064644 | 20077003 | 20077642 | ? / ? |
|  |  | GRMZM2G151863 | 20123081 | 20127252 | ? / membrane |
|  |  | GRMZM2G146514 | 20203138 | 20224156 | nucleic acid binding / ? |
|  |  | AC211652.4_FG001 | 20421666 | 20422166 | ? / ? |
|  |  | AC211652.4_FG002 | 20429258 | 20434163 | protein binding / ? |
|  |  | AC211652.4_FG003 | 20470964 | 20472435 | chitin catabolic process |
|  |  | GRMZM2G112795 | 20529042 | 20532586 | ? / cytosol |
|  |  | GRMZM2G112894 | 20534915 | 20536583 | polysaccharide binding / ? |
|  |  | GRMZM2G101446 | 20566051 | 20572790 | SKP1-like protein 1A / ? |
|  |  | **GRMZM2G171420** | **20758772** | **20762634** | **Unknown** |
|  |  | GRMZM2G171394 | 20762995 | 20764457 | Rapid alkalinization factor |
|  |  | GRMZM2G078143 | 20795669 | 20799891 | Glycine hydroxymethyltransferase / Plasma membrane |
|  |  | GRMZM2G477533 | 20943684 | 20945224 | Putative MYB DNA-binding protein / ? |
|  |  | AC209029.2_FG002 | 20960987 | 20963725 | transferase activity / ? |
|  |  | GRMZM5G833945 | 21073375 | 21074122 | ? / ? |
|  |  | GRMZM2G015295 | 21158461 | 21161610 | methylation-dependent chromatin silencing / plasma membrane |
|  |  | GRMZM2G003970 | 21243636 | 21244649 | galactosyltransferase activity / membrane |
|  |  | GRMZM2G397687 | 21334901 | 21335970 | nutrient reservoir activity / ? |
|  |  | GRMZM2G097135 | 21342654 | 21344213 | chaperone binding / ? |
| 1 | 110914351 | GRMZM2G073026 | 110381642 | 110385472 | structural constituent of ribosome / ribosome |
|  |  | GRMZM2G088765 | 110499018 | 110501408 | oxidation-reduction process / ? |
|  |  | GRMZM2G333641 | 110644724 | 110647283 | protein binding / ? |
|  |  | **GRMZM2G368632** | **110921054** | **110923633** | **Protein serine/threonine kinase activity** |
|  |  | GRMZM2G084825 | 110955450 | 110958921 | Protein serine threonine kinase activity |
|  |  | GRMZM2G154216 | 110981685 | 110983586 | Transferase activity of acyl groups (no amino-acyl) |
|  |  | GRMZM2G703714 | 111050305 | 111050940 | ? / ? |
|  |  | GRMZM2G703716 | 111053776 | 111054411 | ? / ? |
|  |  | GRMZM2G703718 | 111073040 | 111073675 | ? / ? |
|  |  | GRMZM2G703720 | 111075297 | 111075932 | ? / ? |
|  |  | GRMZM2G435244 | 111134940 | 111139069 | triose-phosphate isomerase activity / ? |
|  |  | GRMZM2G343916 | 111208878 | 111231121 | ? / ? |
|  |  | GRMZM2G172081 | 111279177 | 111282013 | protein kinase activity / ? |
|  |  | GRMZM2G172098 | 111285198 | 111288427 | ? / ? |
| 5 | 27857856 | GRMZM2G477683 | 26991391 | 26995651 | transferring acyl groups / ? |
|  |  | GRMZM2G162544 | 27073108 | 27079228 | SNAP receptor activity / trans-Golgi network |
|  |  | GRMZM2G117963 | 27080630 | 27082543 | ? / ? |
|  |  | GRMZM2G174145 | 27119181 | 27122602 | pyridoxal phosphate binding / ? |
|  |  | GRMZM2G341036 | 27150769 | 27152027 | ? / ? |
|  |  | GRMZM2G102862 | 27218204 | 27224147 | protein kinase activity / ? |
|  |  | GRMZM2G001887 | 27413180 | 27416238 | regulation of transcription / ? |
|  |  | GRMZM5G892700 | 27532546 | 27537658 | ? / ? |
|  |  | GRMZM5G800835 | 27584416 | 27585178 | ? / ? |
|  |  | GRMZM2G079343 | 27614332 | 27617077 | zinc ion binding / ? |
|  |  | GRMZM5G864239 | 27663006 | 27663473 | ? / ? |
|  |  | GRMZM2G105065 | 27700864 | 27702756 | ? / ? |
|  |  | **GRMZM2G405090** | **27787706** | **27788723** | **Sequence regulator** |
|  |  | GRMZM2G159399 | 27964682 | 27969438 | response to hormone / nucleus |
|  |  | GRMZM5G868641 | 28083678 | 28084786 | ? / ? |
|  |  | GRMZM2G462081 | 28106280 | 28108028 | ? / ? |
|  |  | GRMZM2G100360 | 28129736 | 28132119 | ? / nucleus, cytosol |
|  |  | GRMZM5G873681 | 28214819 | 28216875 | ? / ? |
|  |  | GRMZM2G068566 | 28221303 | 28223664 | ? / ? |
|  |  | GRMZM2G369939 | 28228844 | 28231675 | RNA metabolic process / nucleus |
|  |  | GRMZM5G840703 | 28327321 | 28327875 | electron transport chain / ? |
|  |  | GRMZM5G841419 | 28340737 | 28341082 | ? / ? |
|  |  | AC233894.1_FG005 | 28353805 | 28355145 | ? / ? |
|  |  | GRMZM2G438755 | 28464122 | 28466705 | ? / ? |
|  |  | GRMZM5G813047 | 28541908 | 28542533 | ? / ? |
| 5 | 27247368 | GRMZM2G013082 | 26463616 | 26466038 | oxidation-reduction process / ? |
|  |  | GRMZM2G428356 | 26614237 | 26617409 | ? / ? |
|  |  | GRMZM2G048559 | 26674856 | 26678303 | cell wall macromolecule catabolic process / ? |
|  |  | GRMZM2G048435 | 26679940 | 26708527 | ? / ? |
|  |  | GRMZM2G173978 | 26785531 | 26795423 | cell redox homeostasis / ? |
|  |  | GRMZM2G174140 | 26984937 | 26987030 | ? / ? |
|  |  | GRMZM2G477683 | 26991391 | 26995651 | transferring acyl groups / ? |
|  |  | GRMZM2G477688 | 26993090 | 26995197 | ? / ? |
|  |  | GRMZM2G477694 | 26998356 | 27013206 | protein binding / ? |
|  |  | GRMZM2G162544 | 27073108 | 27079228 | SNAP receptor activity / trans-Golgi network |
|  |  | GRMZM2G117963 | 27080630 | 27082543 | ? / ? |
|  |  | GRMZM2G174145 | 27119181 | 27122602 | pyridoxal phosphate binding/ ? |
|  |  | GRMZM2G341036 | 27150769 | 27152027 | Unknown |
|  |  | **GRMZM2G102862** | **27218204** | **27224147** | **Protein kinase activity** |
|  |  | GRMZM2G001887 | 27413180 | 27416238 | regulation of transcription / ? |
|  |  | GRMZM5G892700 | 27532546 | 27537658 | ? / ? |
|  |  | GRMZM5G800835 | 27584416 | 27585178 | ? / ? |
|  |  | GRMZM2G079343 | 27614332 | 27617077 | zinc ion binding |
|  |  | GRMZM5G864239 | 27663066 | 27663473 | ? / ? |
|  |  | GRMZM2G105065 | 27700864 | 27702756 | ? / ? |
|  |  | GRMZM2G405090 | 27787706 | 27788723 | ? / ? |
|  |  | GRMZM2G159399 | 27964682 | 27969438 | regulation of transcription / nucleus |
| 7 | 153421340 | GRMZM2G021233 | 152614901 | 152621652 | DNA binding / ? |
|  |  | GRMZM2G045660 | 152714822 | 152716896 | signal transduction / intracellular |
|  |  | GRMZM2G044044 | 152721826 | 152729179 | protein binding / ? |
|  |  | GRMZM2G342985 | 152726925 | 152728424 | ? / ? |
|  |  | GRMZM2G342895 | 152729928 | 152735818 | ? / ? |
|  |  | GRMZM2G121546 | 152740409 | 152778847 | prephenate dehydratase activity / ? |
|  |  | GRMZM2G108849 | 152808839 | 152813057 | cysteine-type endopeptidase activity / vacuola |
|  |  | GRMZM2G108874 | 152813224 | 152817051 | ? / ? |
|  |  | GRMZM2G049151 | 152843404 | 152844283 | ? / ? |
|  |  | GRMZM2G048987 | 152856122 | 152861721 | regulation of transcription / ? |
|  |  | AC211764.3_FG010 | 152863848 | 152864372 | ? / ? |
|  |  | GRMZM2G513222 | 152867472 | 152868074 | ? / ? |
|  |  | GRMZM2G048907 | 152868671 | 152870546 | photosynthetic electron transport in photosystem II / thylakoid membrane |
|  |  | GRMZM2G048819 | 152870773 | 152892049 | 3'-5' exonuclease activity / intracellular |
|  |  | GRMZM2G154489 | 152988408 | 152989553 | calcium ion binding / ? |
|  |  | AC212216.4_FG003 | 152989672 | 152992467 | nucleic acid binding / ? |
|  |  | GRMZM2G154574 | 152997411 | 153005288 | telomere maintenance in response to DNA damage / ? |
|  |  | GRMZM2G117642 | 153136076 | 153138811 | deaminase activity / thylakoid |
|  |  | GRMZM2G449709 | 153211794 | 153213046 | ? / ? |
|  |  | GRMZM2G148272 | 153219518 | 153220328 | ? / ? |
|  |  | GRMZM2G075828 | 153256118 | 153260594 | drug transmembrane transporter activity / membrane |
|  |  | GRMZM2G075794 | 153288576 | 153290674 | ? / ? |
|  |  | GRMZM2G127510 | 153320097 | 153324724 | Nucleotide binding |
|  |  | GRMZM2G127499 | 153326363 | 153329791 | Unknown |
|  |  | GRMZM2G429396 | 153330870 | 153331777 | Response to high light intensity / Cytoplasm |
|  |  | GRMZM2G124794 | 153413756 | 153414250 | Deoxyribodipyrimidine photo-lyase activity |
|  |  | GRMZM2G423478 | 153416453 | 143416869 | Unknown |
|  |  | **GRMZM2G180027** | **153457846** | **153464555** | **Unknown** |
|  |  | GRMZM2G480480 | 153464547 | 153475981 | Protein binding |
|  |  | GRMZM2G180080 | 153485164 | 153487062 | Nucleotide binding |
|  |  | GRMZM2G180082 | 153490387 | 153492351 | Oxidoreductase activity, acting on paired donors, with incorporation or reduction of molecular oxygen |
|  |  | GRMZM2G112374 | 153572197 | 153572738 | ? / ? |
|  |  | GRMZM2G177300 | 153595348 | 153597540 | ? / ? |
|  |  | GRMZM2G150791 | 153608379 | 153609558 | isopentenyl diphosphate biosynthetic process / chloroplast stroma |
|  |  | GRMZM2G451672 | 153609824 | 153619217 | jasmonic acid biosynthetic process / ? |
|  |  | GRMZM2G360949 | 153640760 | 153644392 | ? / ? |
|  |  | GRMZM2G167591 | 153692969 | 153695938 | ? / ? |
|  |  | GRMZM5G873431 | 153740060 | 153740854 | ? / ? |
|  |  | GRMZM2G415529 | 153745113 | 153759535 | ATPase activity / membrane |
|  |  | GRMZM2G027344 | 153769742 | 153774536 | small GTPase mediated signal transduction / intracellular |
|  |  | GRMZM2G009412 | 153856520 | 153858758 | structural constituent of ribosome / ribosome |
|  |  | GRMZM2G009223 | 153860994 | 153863453 | transporter activity / integral component of membrane |
|  |  | GRMZM2G009144 | 153881458 | 153882749 | xylan biosynthetic process / ? |
|  |  | GRMZM2G009103 | 153894302 | 153896666 | transmembrane transporter activity / membrane |
|  |  | GRMZM2G051709 | 153985839 | 153988165 | transmembrane transporter activity / membrane |
|  |  | GRMZM2G056627 | 154158439 | 154160973 | cellulose microfibril organization / anchored component of membrane |
