# Supplementary material for: Association mapping for cold tolerance in two large maize inbred panels
Source: BMC Plant Biol. 2016 Jun 6;16:127. doi: 10.1186/s12870-016-0816-2 (PMC4895824; doi:10.1186/s12870-016-0816-2)
Supplement: Additional file 4: Table S4. — Flint inbred lines with the favorable allele for the SNPs significantly associated to cold tolerance-related traits and allele composition for the significant SNPs with highest level of signification from the testcross trials of flint inbreds. (DOCX 36 kb) [file 12870_2016_816_MOESM4_ESM.docx]

| Table S4. Flint inbred lines with the favorable allele for the SNPs significantly associated to cold tolerance-related traits, favorable allele composition of haplotype groups, and allele composition for the significant SNPs with highest level of signification from the testcross trials of flint inbreds. | | | | | | | | | | |
| --- | --- | --- | --- | --- | --- | --- | --- | --- | --- | --- |
|  |  | Significant SNPs. Inbreds *per se* | | | | | | Significant SNPs. Testcrosses | | |
| Group | Inbred | 2238^a^ ΦPSII  ^c^CCA | 16497 Days to emergence  ^c^TTAG | 19550 ΦPSII  ^c^AAGA | 25317  Early vigor  ^c^T | 25329  Early vigor  ^c^T | 37073  Early vigor  ^c^TT | 13270  G | 38019  A | 39863  C |
| N_NF | C105 | CCA | -T-- | -AGA | T | T | TT | G | - | C |
| NF | CH19-1 | C-- | TTAG | AAGA | T | T | -T | G | - | C |
| N_NF | CH36^b^ | CCA | TTAG | -AG- | T | T | -T | G | A | C |
| NF_D171 | D171 | CCA | TTAG | -AG- | T | T | -T | G | - | C |
| N_NF | EP32 | CCA | TTAG | -AG- | T | T | TT | - | - | - |
| N_NF | EZ33 ^b^ | CCA | TTAG | -AGA | T | T | TT | G | A | C |
| NF_D171 | F02803 | CCA | TTAG | -AG- | T | T | -T | G | A | - |
| NF_D171 | F363 | CCA | TTAG | -AG- | T | T | -T | G | - | C |
| Z_NG | FV11 | C-- | TTAG | -AGA | T | T | TT | G | A | - |
| NF | FV18 | C-- | TTAG | -AGA | T | T | -T | G | A | - |
| N_NF_FV7 | FV286 | CCA | TTAG | -AGA | T | T | -T | G | A | - |
|  | FV361 | CCA | TTAG | -AG- | T | T | -T | G | - | C |
| N_NF | FV70 | C-- | TTAG | -AG- | T | T | TT | G | A | - |
| N_NF | FV71 | C-- | TTAG | -AG- | T | T | TT | G | - | C |
| N_NF | Ia2132 ^b^ | CCA | TTAG | -AGA | T | T | TT | G | A | C |
| NF | PLS41 | CCA | TTAG | -AG- | T | T | -T | G | - | C |
| NF | PLS42 | CCA | TTAG | -AG- | T | T | -T | G | - | C |
| NF_FV7 | UH1118 | CCA | TTAG | -AG- | T | T | -T | G | A | - |
| NF_D171 | UH1494 ^b^ | CCA | TTAG | -AG- | T | T | -T | G | A | C |
| Z_Mixed_FV7 | UH3056 | CCA | TTAG | -AG- | T | T | -T | G | - | - |
| NF_D171 | UH5222 | C-- | TTAG | -AG- | T | T | -T | G | - | C |
| NF_D171 | UH5248 | CCA | TTAG | -AG- | T | T | -T | G | - | C |
| NF_D171 | UH5271 | CCA | TTAG | -AG- | T | T | -T | G | - | C |
| NF_D171 | UHF016 | CCA | TTAG | -AG- | T | T | -T | G | - | C |
| NF_D171 | UHF027 | CCA | TTAG | -AG- | T | T | -T | G | - | - |
| NF_D171 | UHF043 | CCA | TTAG | -AG- | T | T | -T | G | - | - |
| NF_D171 | UHF050 | CCA | TTAG | -AG- | T | T | -T | G | - | C |
| NF_D171 | UHF070 | CCA | TTAG | -AG- | T | T | -T | G | - | - |
| NF_D171 | UHF082 | CCA | TTAG | -AG- | T | T | -T | G | - | - |
| NF_D171 | UHF084 | CCA | TTAG | -AG- | T | T | -T | G | - | - |
| NF_D171 | UHF091 | CCA | TTAG | -AG- | T | T | -T | G | - | - |
| NF_D171 | UHF093 | CCA | TTAG | -AG- | T | T | -T | G | - | - |
| NF_D171 | UHF098 | CCA | TTAG | -AG- | T | T | -T | G | - | - |
| NF_D171 | UHF105 | CCA | TTAG | -AG- | T | T | -T | G | - | - |
| NF | UHFF0721H | C-- | TTAG | AAGA | T | T | -T | G | - | C |
| N_NF | UHL003 | C-- | TTAG | -AG- | T | T | -T | - | A | C |
|  | UHL010 | C-- | TTAG | --G- | T | T | -T | - | - | C |
| NF_D171 | UHL016 | CCA | TTAG | AAGA | T | T | -T | G | - | C |
| NF_D171 | UHL021 | CCA | TTAG | -AG- | T | T | -T | - | - | C |
| NF_D171 | UHL031 | CCA | TTAG | -AG- | T | T | -T | - | A | C |
| NF_D171 | UHL038 | CCA | TTAG | -AG- | T | T | -T | - | - | C |
| NF_D171 | UHL048 | CCA | TTAG | -AG- | T | T | -T | G | - | - |
| NF_D171 | UHL054 | CCA | TTAG | -AG- | T | T | -T | - | - | C |
| NF_D171 | UHL058 | CCA | TTAG | -AG- | T | T | -T | G | - | - |
| NF_D171 | UH006 | CCA | TTAG | -AG- | T | T | -T | G | - | - |
| NF_D171 | UH007 | CCA | TTAG | -AG- | T | T | -T | G | - | C |
| NF | YUBR6 | CCA | TTAG | -AG- | T | T | -T | G | - | C |
| ^a^ SNP site (see Table 2 for precise information) ^b^ inbred with favorable allele for all QTLs from the inbreds *per se* evaluated under cold conditions and the three QTLs with higher significant level from the testcross evaluation under cold conditions.  ^c^ Favorable alleles within each haplotype. The significant SNP is underlined | | | | | | | | | | |
