# Supplementary material for: Association mapping for cold tolerance in two large maize inbred panels
Source: BMC Plant Biol. 2016 Jun 6;16:127. doi: 10.1186/s12870-016-0816-2 (PMC4895824; doi:10.1186/s12870-016-0816-2)
Supplement: Additional file 5: Table S5. — Dent inbred lines with the allele composition for the SNPs significantly associated to cold tolerance-related traits and allele composition for the significant SNPs with highest level of signification from the testcross trials of flint inbreds. Data: Best linear unbiased estimates (BLUEs) of early growth-related traits used for association analyses for cold tolerance traits in two association panels of maize inbred lines evaluated per se and hybrids under control and cold conditions. (DOCX 30 kb) [file 12870_2016_816_MOESM5_ESM.docx]

| Table S5. Dent inbred lines with the allele composition for the SNPs significantly associated to cold tolerance-related traits, favorable allele composition of haplotype groups, And allele composition for the significant SNPs with highest level of signification from the testcross trials of flint inbreds. | | | | | | |
| --- | --- | --- | --- | --- | --- | --- |
|  |  | Significant SNPs. Inbreds *per se* | | | Significant SNPs. Testcrosses | |
| Group | Inbred | 3075  Early vigor  ^C^CCGGGCTA | 4871  SPAD ^C^ATTC | 22573  SPAD  ^C^G | 18094  G | 25302  T |
|  | A347 | -CGGGC-A | ATTC | G | - | T |
| Mo17 | AS5707 | CC------ | ATTC | G | - | - |
|  | B103 | CC------ | ATTC | G | G | - |
| Mixed | B110 | -CGG-CTA | ---C | G | - | T |
| NG | B112 | -CGG-CTA | ---C | G | - | T |
| Stiff_Stalk_B73 | B37 | -CGG-CTA | ATTC | - | G | T |
| NG | B99 | -CGGACTA | ---C | G | - | T |
| Mixed | EP29 | -CGG-CTA | -T-C | G | - | T |
| M13 | EP51 | -CGG-CTA | ATTC | - | - | - |
| M13 | EZ11A | -CGGGC-A | ATTC | G | G | T |
| Mixed | EZ46 | -CGGGC-A | ATTC | G | G | T |
| Mixed | EZ47 | -CGG-CTA | ATTC | G | - | T |
| Mixed | EZ5 | C------- | ATTC | G | - | - |
| Mixed | F608 | -CGG-CTA | -T-C | G |  | - |
| M13 | F618 | -CGG-CTA | ATTC | - | G | T |
| OnI_F252 | F7025 | -CG--C-A | ATTC | G | - | T |
| Mixed | F838 | -CGG-CTA | -T-C | G | - | T |
| M13 | F904 | CC------ | ATTC | G | - | - |
| OnI_F252 | F918 | -CGG-CTA | ATTC | - | G | T |
| Stiff_Stalk | F924 | -CGG-CTA | ATTC | G | - | T |
| Mixed | FV317 | -CG--C-A | ATTC | G | G | T |
| Iodent_Ph207 | FV335 | -CGGGC-A | ATTC | G | - | T |
| M13 | LH82 | -CGG-CTA | -T-C | G | - | T |
| Mixed | LH85 | -CGGGC-A | ATTC | G | - | - |
| Mo17 | LH93 | -CGG-CTA | -T-C | - | - | T |
| M13 | N6 | -CGG-CTA | -T-- | G | G | T |
| Mo17 | NC258 | C------- | ATTC | G | - | T |
| M13 | NC262B | -CGG-CTA | -T-- | G | - | T |
| Mixed | NC290 | -CGG-CTA | -T-- | G | - | T |
| M13 | NK807 | -CGG-CTA | ATTC | G | - | T |
| M13 | Oh02 | -CGG-CTA | ATTC | - | - | T |
| M13 | PHB09 | -CGG-CTA | ATTC | - | G | - |
| Stiff_Stalk_B14a | PHG35 | -C---- | ATTC | G | A | T |
| Iodent_Ph207 | PHG80 | -CGGG-CTA | ATTC | - | G | T |
| Mixed | PHJ40 | -CGG-CTA | ATTC | G | - | T |
| Mo17 | PHK29 | CC------ | ATTC | G | - | T |
| M13 | PHK76 | -CGGGC-A | ATTC | G | - | T |
| Mixed | T8 | -CGG-CTA | ATTC | - | G | - |
| OnI_F252 | UH1595 | -CGG-CTA | ATTC | - | - | T |
| Iodent_Ph207 | UH6103 | -CGG-CTA | ---C | G | - | T |
| Iodent_Ph207 | UH6110 | -CGG-CTA | ---C | G | - | T |
| Iodent_Ph207 | UH6132 | -CGG-CTA | ---C | G | - | - |
| Iodent_Ph207 | UHP046 | -CGG-CTA | -T-C | G | G | C |
| Iodent_Ph207 | UHP060 | -CGG-CTA | ---C | G | - | T |
| Iodent_Ph207 | UHP075 | -CGG-CTA | ---C | G | - | C |
| Iodent_Ph207 | UHP087 | -CGG-CTA | -T-C | G | - | T |
| Iodent_Ph207 | UHP104 | -CGG-CTA | ---C | G | - | T |
| Iodent_Ph207 | UH250 | -CGG-CTA | ---C | G | - | T |
| M13 | WH | -CGG-CTA | -T-C | G | - | T |
| ^a^ SNP site (see Table 2 for precise information) with the favorable allele in parenthesis  ^c^ Favorable alleles within each haplotype. The significant SNP is underlined | | | | | | |
